# Programmable Inter‐Droplet Communication by Polymer‐Gated Ion Transport Through Nanopore Lattices

**DOI:** 10.1002/smsc.70352

**Published:** 2026-07-29

**Authors:** Marius Kirsch, Agustin D. Pizarro, Martín G. Bellino, Galo J. A. A. Soler‐Illia, Annette Andrieu‐Brunsen

**Affiliations:** ^1^ Ernst‐Berl Institut für Technische und Makromolekulare Chemie Technische Universität Darmstadt Darmstadt Germany; ^2^ Instituto de Nanosistemas Escuela de Bio y Nanotecnologías (INS‐EByN‐UNSAM‐CONICET) San Martín Argentina; ^3^ Instituto de Nanociencia y Nanotecnología (CNEA‐CONICET) San Martín Buenos Aires Argentina

**Keywords:** chemical engineering, imbibition, materials science, mesoporous material, mesoporous silica, nanopore, nanotechnology, polyelectrolyte, polymerization

## Abstract

Owing to their hydrophilicity and pronounced capillary pressures, mesoporous platforms enable isotropic or multidirectional fluid transport. Based on aqueous salt solution droplets, we investigate nanopore‐driven ion transport control for tailorable droplet communication *via* a mesoporous surface. Droplets were placed on a mesoporous silica thin film, separated by a polyelectrolyte (PEL) barrier. The PEL‐barrier was incorporated into the mesoporous matrix by automated direct‐laser‐writing and a grafting‐from SI‐PET‐RAFT polymerization. Permselective transport‐regulated barrier breakthrough times were used to compare aqueous BaCl_2_, MgSO_4_, MgCl_2_, and NaCl solutions. By tuning the barrier widths, the full range of the polyelectrolyte barrier functions from ion‐selective flow regulation to complete fluid retention becomes attainable. Ion‐specific barrier interactions lead to characteristic imbibition dynamics beyond the barrier, involving advancing imbibition toward a steady state in the case of favored barrier permeation, or hindered and oscillating imbibition in the case of disfavored barrier permeation. The ionic selectivity further allows the emergence of control of chemical events such as precipitation reactions in nanopores by the spatial localization of droplets according to the barrier site. The insights into polyelectrolyte‐regulated ion transport during imbibition processes bring new tools for the development of compartmentalized and reconfigurable communication between distant fluid reservoirs through nanostructured coatings.

## Graphical abstract

Laser‐written polyelectrolyte barriers were applied for regulating imbibition and ion transport through silica mesopores between droplet compartments. Specific imbibition sequences and precipitation readouts indicated cation‐selective transport across the barriers. Depending on salt identity, barrier, and experimental parameters, ionic transport ranged from complete ion retention to unhindered permeation through the barrier.

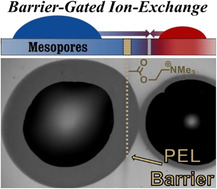

## 1 Introduction

Capillary phenomena govern liquid uptake, movement, and solute transport, driving processes such as plant hydration [1], soil wetting [2], or liquid permeation through membranes [3, 4]. Especially, mesoporous materials (i.e., pore diameter between 2 and 50 nm) give rise to capillarity‐driven liquid uptake and multidirectional transport, a process generally referred to as imbibition [5]. By enabling liquid transport between well‐defined chemically complementary compartments connected through a mesoporous network, imbibition may provide a relevant model basis for the future development of artificial‐cell architectures involving chemical communication between spatially separated compartments, a central challenge in synthetic biology [6], as well as in nanoreactors or ion exchange between electrodes [4]. In practice, the liquid transport becomes macroscopically observable for instance by depositing two droplets as independent compartments on a mesoporous thin film where the forming imbibition ring (annulus) around the droplet compartment connects both droplets through the underlying mesoporous network, thereby allowing for *droplet communication* [7, 8]. By harnessing the droplets as chemically complementary reactors [8], droplet communication holds great promise to explore signal transduction mechanisms fundamental in biointeractions to design droplet frameworks as active matter systems [7, 9]. From a broader perspective, imbibition in mesoporous materials provides a model platform for studying liquid transport through porous separation layers, with surface functionalization paving the way for both transport control and ionic gating platforms [8, 10, 11, 12, 13, 14, 15, 16, 17, 18, 19].

**FIGURE 1:**
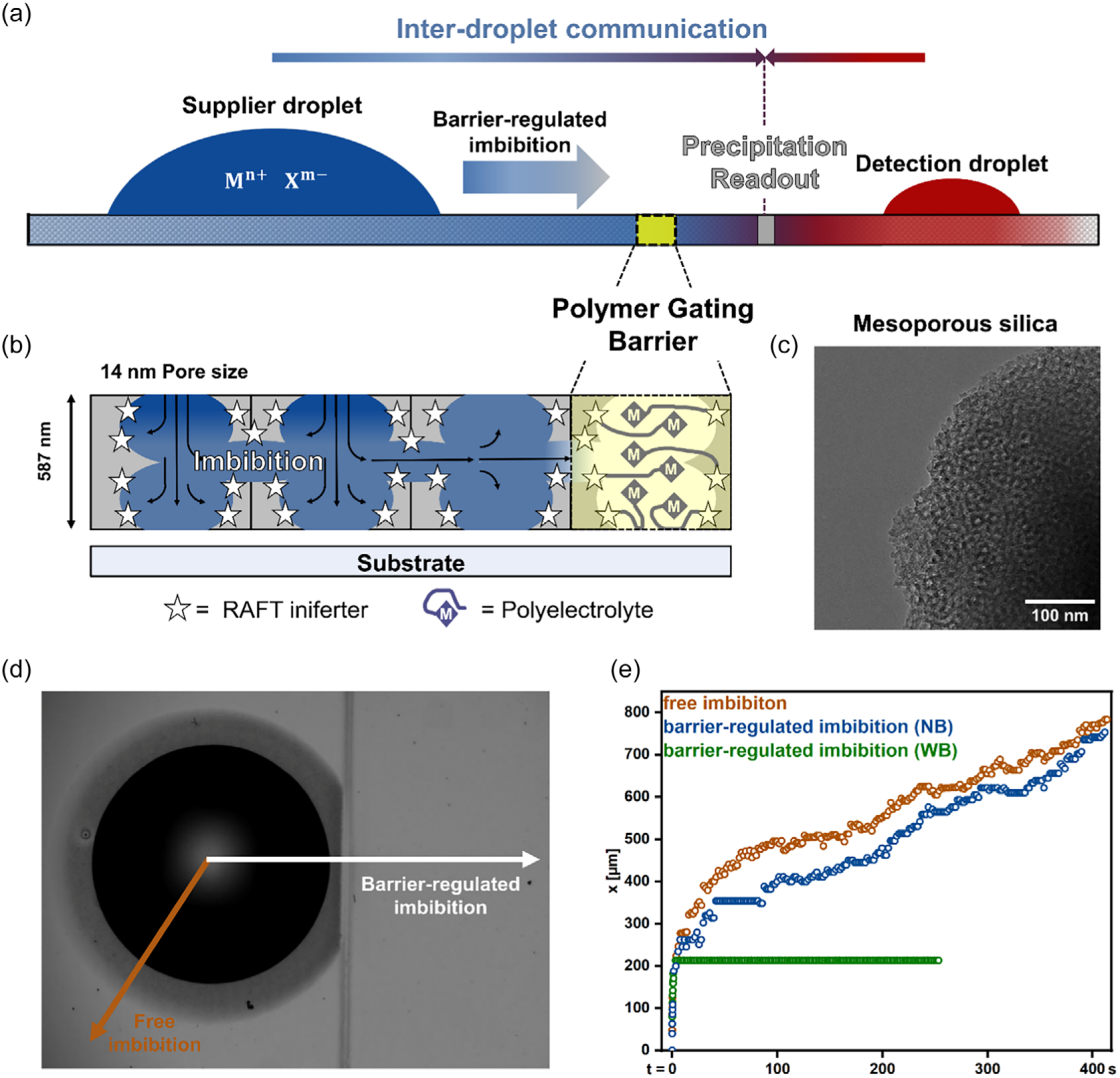
(a) Schematic illustration of *barrier‐regulated imbibition* and *droplet communication* through functionalized mesoporous silica thin films, originating from an aqueous (aq.) salt solution (supplier) droplet; the imbibition leads toward a polyelectrolyte barrier, targeting barrier permeation and in the case of droplet communication contact with the imbibition ring of the detection droplet; the contact of both imbibition rings is indicated by a precipitation readout. (b) Schematic illustration of the mesoporous thin film structure, indicating imbibition advancement through the RAFT iniferter functionalized network of pores toward the surface‐grafted polyelectrolyte barrier; (c) transmission electron microscopy image of a mesoporous silica thin film; (d) droplet imbibition image in top‐view, showing the expansion of the annular imbibition ring (dark gray) originating from the droplet (black) as well as the contact line of the imbibition ring with the barrier (light to dark gray line); *barrier‐regulated imbibition* (advancement direction indicated by the white arrow) advances perpendicular to the barrier whereas *free imbibition* (advancement direction indicated by the red arrow) advances in averted direction to the barrier (the tested barriers vary in widths and are accordingly named *narrow* and *wide barrier*); (e) characteristic tracking of fluid front advances to illustrate the distinct imbibition progress (stop‐and‐go occurrence) generated from the narrow barrier.

As the physical basis of transport under nanoconfinement, liquid imbibition in porous materials is driven by capillary pressures and opposed by viscous resistance [5]. To describe the imbibition in air‐exposed mesoporous thin films, an evaporation‐related contribution was included in the classical Lucas–Washburn equation [20]. In contrast to the sustained imbibition advancement typical for closed systems, this model captures the counter‐balance between capillarity and evaporation that leads to a *steady state* of constant imbibition widths [20]. This behavior is characteristic for pure water and dilute aqueous salt solutions imbibing into hydrophilic mesoporous thin films and is also observed upon salt precipitation [21, 22, 23]. In contrast, at higher ion concentrations (*C*_salt_ > 1 mmol L^−1^), the imbibition dynamics may evolve over time due to evaporation‐induced changes in the composition of the fluid confined within the mesopores, a behavior previously rationalized using phenomenological models [21, 22]. The detailed effect of such compositional variations is yet unclear but likely involves osmotic pressure contributions between the thin‐film‐deposited droplet and the surrounding annular imbibition zone as well as specific ion effects (Figure 1) [21, 22].

The inherent negative surface charge of silica in neutral pH naturally positions ions as cargo species for transport processes through mesoporous silica (MPS) networks [24, 25]. Due to the deprotonation of silanol groups at pH values above silica's isoelectric point [25], silica mesopores strongly favor the transport of cations over anions, as evidenced by cyclic voltammetry involving cationic and anionic redox probes at pH 5 [26]. The ion affinity of transparent silica mesopores is readily tunable by surface functionalization [12, 13, 14, 15, 20], with a growing emphasis on light‐induced and surface‐initiated reversible‐addition‐fragmentation (RAFT) chain‐transfer polymerization protocols, enabling the grafting of ion‐responsive polyelectrolytes from the mesoporous wall [27, 28, 29, 30, 31]. Based on electrostatic interactions with migrating ions, surface‐bound polyelectrolytes function as ion gates, enabling cation and anion selective permeation through the mesoporous matrix [12, 13, 14, 31]. Electrostatic gating of ion transport through polyelectrolyte‐functionalized mesopores has been demonstrated for stimuli including light [10, 32], temperature [33], electrical potential [34], pH change [13], and reactive small molecules [35, 36]. Furthermore, pH‐responsive polyacids or polybases [31], as well as ion‐complexing polyelectrolytes [37] have been employed as surface‐active functions to establish a switchable and thus alternating anion or cation accessibility of mesopores. The gradual increase of polymer amounts and thus pore filling by surface‐initiated, controlled polymerization techniques enables the generation of tunable charge densities within mesoporous matrices [14, 38, 39], thereby providing a versatile platform for regulating diffusion and transport processes of charged and uncharged solute species.

Programming intrapore charge densities envisions a promising strategy to adapt the mesopore environment to the physicochemical demands of the permeating cargo, ultimately laying the foundation for selective and efficient transport up to gating applications. Owing to its tunable irradiation energy doses and local precision, the recently developed Direct Laser Writing (DLW) technique permits the incorporation of functional polyelectrolyte arrays into transparent mesoporous thin films with local precision [28, 40], thus providing a robust pathway to coherent functional microstructures within the mesopores for transport control. Despite the availability of spatially precise photopolymerization strategies, the role of intrapore polyelectrolyte loading in regulating ionic transport through mesoporous thin films as programmable droplet intercommunication remains unexplored. Specifically, it is worth analyzing to what extent polymer–ion interactions can be exploited to control the permeation of ions under imbibition processes to achieve smart ion intercommunication between different functional regions along mesoporous thin film structures.

Herein, we harness DLW to fabricate polyelectrolyte barriers with well‐defined dimensions between two different regions of MPS thin films. This provides the foundation to conduct localized barrier‐regulated fluid imbibition concomitant with ionic transport between two different droplets placed on the film surface at opposite sites of the polyelectrolyte barrier. By imaging the extent of cationic polyelectrolyte barrier‐regulated imbibition, we explore how ion characteristics, as well as barrier properties and experimental parameters, govern the imbibition and thus modulate the barrier‐gated ion and fluid transport. Building on these insights, we pursue barrier‐regulated droplet communication based on converging imbibition rings by depositing two chemically complementary droplet compartments separated by the barrier. Hence, we achieve modular chemical signaling between both droplets via the underlying mesoporous network, using ions that undergo precipitation reactions as an easily observable macroscopic readout thanks to the transparent nature of the platform [41]. By establishing a new and reproducible way to regulate spontaneous transport between droplet compartments through a well‐defined spatial polymer–mesopore architecture, our results open a route toward the rational design of inter‐droplet fluidic systems, with DLW‐generated polyelectrolyte microbarriers as a building block for future smart optofluidic circuits.

## 2 Results and Discussion

MPS thin films modified with polymers were used as a nanofluidic platform (Figure 1). The MPS were fabricated by dip‐coating using the well‐established Evaporation‐Induced Self‐Assembly (EISA) processes [42] and exhibit an average pore diameter of 11 ± 2 nm (see Figure 1c and Figure S1), a contact angle (CA) of 15° before functionalization, a layer thickness of 587 ± 6 nm, and a porosity of 48 ± 2% (see experimental section for details). The amphiphilic block copolymer Pluronic F127 was employed as a mesopore template undergoing micellization during the EISA process at a template loading of 104.4 mg F127 per mmol of SiO_2_ (preparation of the related sol–gel precursor shown in the experimental section), associated with a 3D‐interconnected mesopore network with local pore order according to a literature report using a very similar template loading [43]. Imbibition within such networks is well described by the evaporation‐modified Lucas–Washburn equation, an effective‐parameter model in which pore geometry, connectivity, and tortuosity are lumped into effective capillary parameters related to pore diameter and porosity [20]. By surface‐grafting of the photoiniferter 4‐(trimethoxysilyl)benzyl diethylcarbamodithioate) (SBDC), the MPS thin films were rendered applicable for the light‐induced surface‐initiated photopolymerization of the monomer [2‐(methacryloyloxy)ethyl]trimethylammonium chloride (META‐Cl), thereby enabling the implementation of DLW‐enabled PMETA‐Cl polyelectrolyte barriers into the film. In SBDC functionalized MPS thin films, exhibiting an increased macroscopic static CA of 46°, a steady‐state water imbibition ring was observed with a 206.7 ± 19.3 µm width (Figure S2). This significantly reduced imbibition width, in contrast to unfunctionalized MPS thin films (CA = 20°) with maximum imbibition widths of 500 µm [23], illustrates the dependence of water imbibition on surface wettability due to the addition of the nonpolar surface‐active SBDC groups that reduce the maximum imbibition widths as discussed in our previous work [23]. To investigate the influence of polyelectrolyte barriers on ionic transport between two polyelectrolyte‐barrier separated droplet compartments, PMETA‐Cl barriers of different width were DLW‐prepared into the MPS thin films following a previously established protocol [28, 40].

### 2.1 Experimental Design

Polyelectrolyte barrier‐regulated imbibition experiments (Figure 1) were performed to investigate the effects of a line‐shaped polyelectrolyte barrier on the solution and ion transport (Figure 1a, barrier‐regulated imbibition) and on the ion transport between two droplets separated by the barrier (Figure 1b, droplet communication). In droplet communication experiments (Figure 1b), the larger volume of the 1^st^ droplet ensured liquid transport through the barrier, while the smaller volume of the second droplet was found to be sufficient to induce imbibition ring contact after barrier permeation of the first imbibition ring on the opposite side of the barrier. The presence of the polymer barrier can result in a physical retention of the imbibition ring at the barrier or the permeation of the imbibition ring through the barrier (Figure 1d). The transport between two communicating droplet compartments, containing different salts and being located on opposite sides of the barrier, can be simply analyzed by macroscopically observable precipitation reactions (Figure 1b). To examine barrier effects, barrier‐regulated imbibition (Figure 1d, white arrow), proceeding in a perpendicular direction toward the polyelectrolyte line, is compared to barrier‐unaffected, free imbibition at the droplet side opposite to the barrier. This comparison between barrier‐regulated and free imbibition allows us to reproducibly obtain barrier‐dependent results and barrier‐free controls from the same measurement (Figure 1d, red arrow). PMETA‐Cl was grafted inside MPS thin films by DLW with lateral precision on the µm scale (Figure S3a). The laser beam penetrates through the transparent MPS film from surface to bottom (Figure S3b), leading to well‐defined PMETA‐Cl lines. ZnTPP‐catalyzed, surface‐initiated photoinduced electron/energy transfer reversible addition–fragmentation chain transfer (SI‐PET‐RAFT) polymerization ensured low reaction times of 1 s per polymer spot at a wavelength of 405 nm. Using inter‐spot distances of 10 µm, well below the radius of the laser beam cross section, the DLW‐fabricated polymer spot arrays were condensed into lines, called polymer barriers. With typical lengths of 4 cm and varying widths on the µm scale (Figure S3c–e), PMETA‐Cl polymer barriers were used to separate two droplet compartments deposited onto the MPS thin film on opposite sides. Two barrier designs, distinguished by width ranges of 33 – 50 µm (narrow barrier type–NB) and 83–125 µm (wide barrier type–WB), were produced by applying two different laser powers (Figure S4a). Fluorophore adsorption to the laser‐written polyelectrolyte barriers allowed to visualize the regular shape and widths of the obtained barriers (Figure S3c–d). Barrier widths determined from the corresponding fluorophore images are approximately 7.5‐fold higher than the barrier widths determined by refractive index changes, observed in optical imaging (Figure S3c–d). The discrepancy in barrier widths reflects the different detection limits of the imaging methods, as fluorescence imaging benefits from optical magnification and strong dye‐to‐background contrast, whereas optical imaging primarily resolves the central region of the barrier located near the intensity maximum of the laser beam. Furthermore, fluorescence imaging is much more sensitive to low polymer amounts than ATR‐IR spectroscopy, allowing the detection of polymer distributions below the effective ATR‐IR detection limit [44]. Based on the near‐Gaussian profile of fluorescence‐derived gray value (GV) profile over the NB cross section (Figure S3e), which is consistent with the Gaussian intensity profile of the laser, a gradient of polymer amount toward the center of the barrier typical for DLW is expected in accordance with previous studies in our research group [44].

### 2.2 Ion‐Specific Barrier‐Regulated Imbibition

**FIGURE 2:**
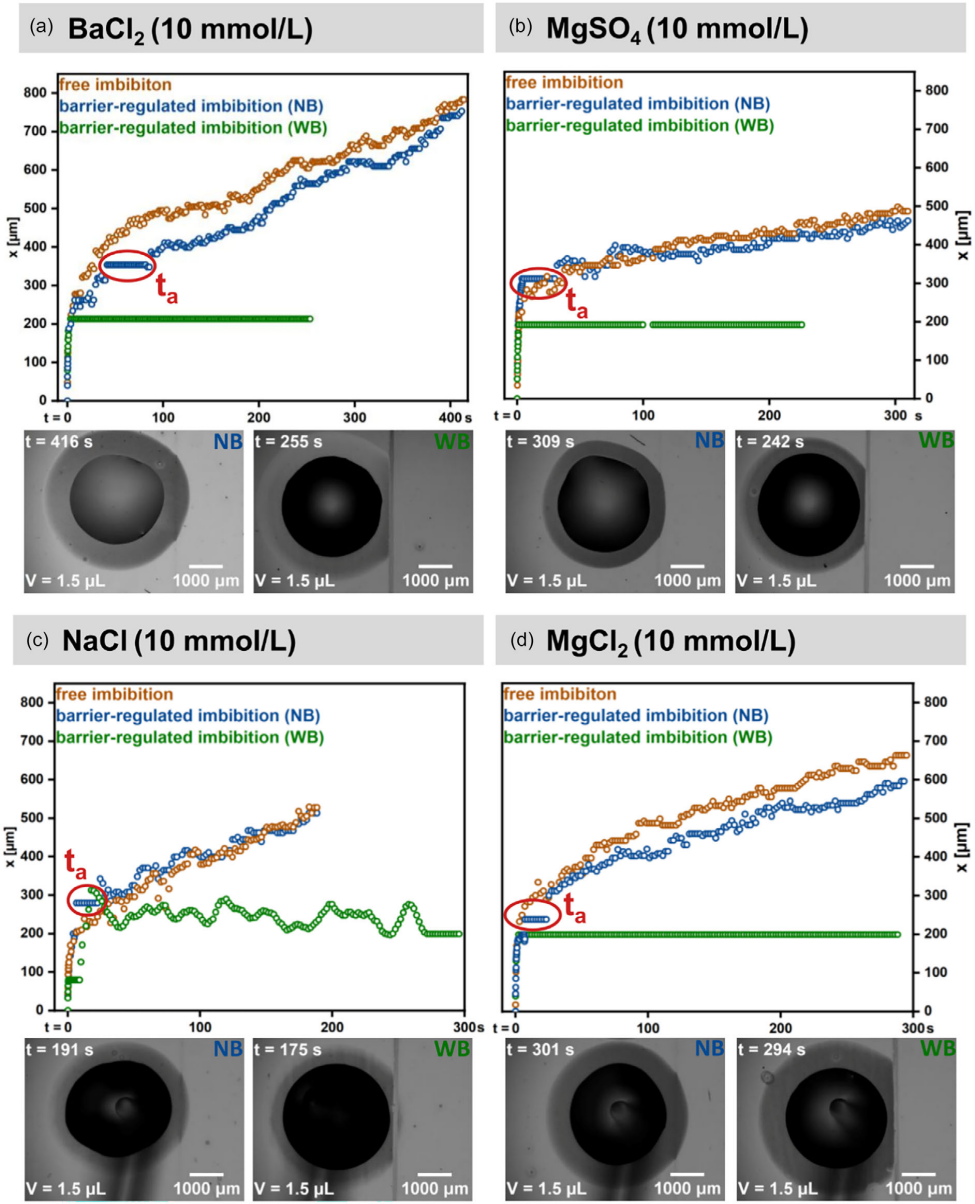
PMETA‐Cl barrier‐regulated droplet imbibition of aqueous salt solutions: temporal evolution of imbibition widths (x) of free imbibition (corresponding to the NB‐regulated imbibition experiments, respectively), NB‐regulated imbibition, and WB‐regulated imbibition, as well as images showing the droplet (black) and imbibition ring (gray annulus) in top‐view before evaporation‐initiated droplet deformation of (a) BaCl_2_, (b) MgSO_4_, (c) NaCl, and (d) MgCl_2_. The barrier breakthrough times are indicated in red in the respective charts.

**FIGURE 3:**
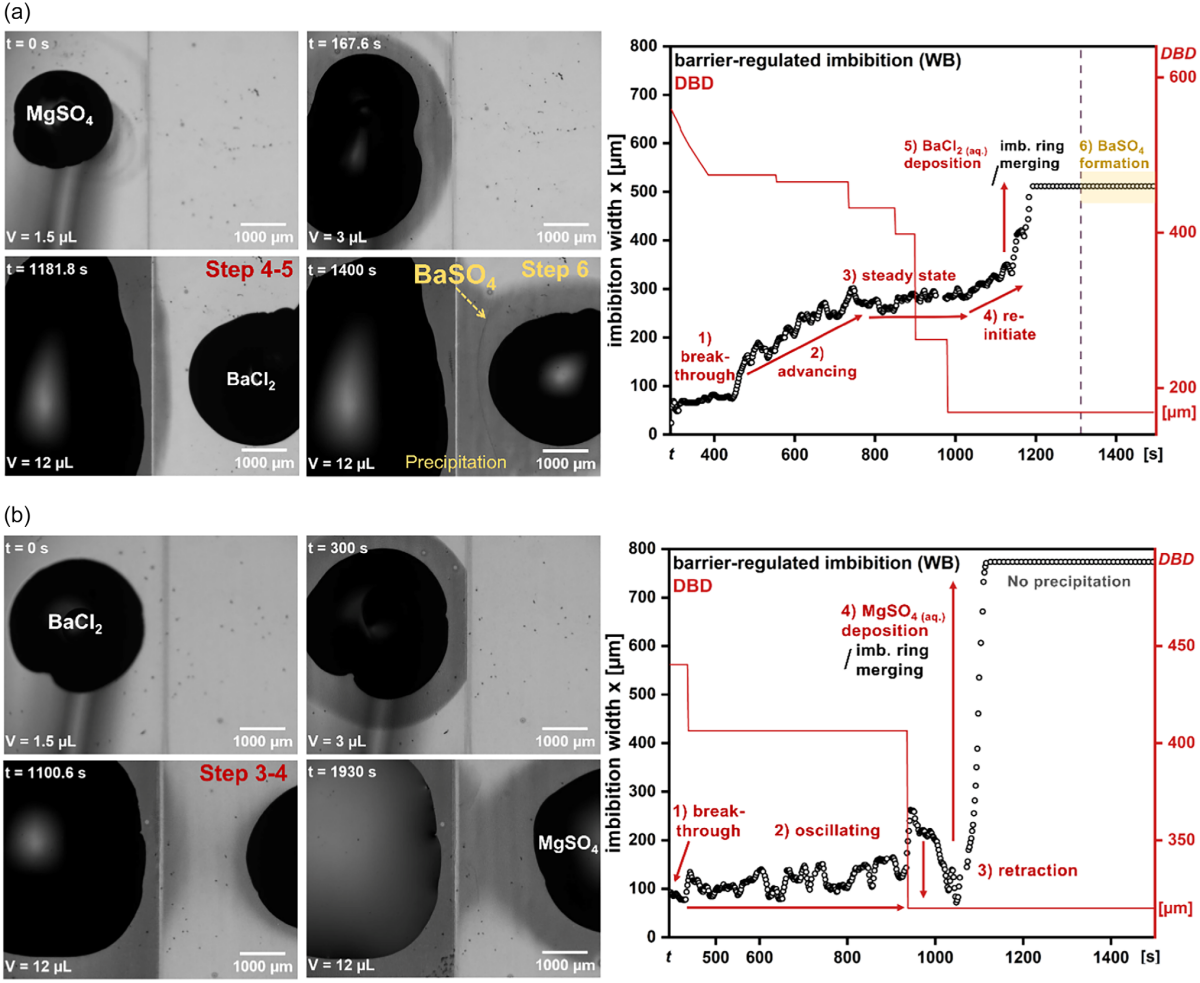
(a) WB‐regulated imbibition of aqueous MgSO_4_ solution and communication between droplets of aqueous MgSO_4_ solution (left, 10 mmol L^−1^, volume 12 µL, deposited first) and aqueous BaCl_2_ solution (right, 10 mmol L^−1^, volume 1.5 µL, deposited after barrier breakthrough of the aqueous MgSO_4_ solution drop), which are separated by the PMETA‐Cl barrier: imaging of the barrier‐regulated imbibition and droplet communication as well as tracking of imbibition width x (smoothed using a 5‐point moving average) upon initial barrier crossing relative to the center of the barrier at an imbibition width of 0, indicating the current DBD which is reduced by an increase of droplet volume during the process; the dashed line indicates the onset of BaSO_4_ precipitation at the contact line of the imbibition (imb.) rings originating from the MgSO_4_ and BaCl_2_ solution droplets; the adjacent color gradient indicates a gradual increase in visible of the precipitate over time. (b) WB‐regulated imbibition of aqueous BaCl_2_ solution and communication between droplets of aqueous BaCl_2_ solution (left, 10 mmol L^−1^, volume 12 µL, deposited first) and aqueous MgSO_4_ solution (right, 10 mmol L^−1^, volume 3 µL, deposited after barrier breakthrough of the aqueous MgSO_4_ solution drop) which are separated by the barrier: imaging of barrier‐regulated imbibition and droplet communication as well as tracking of imbibition width x (smoothed using a five‐point moving average) upon initial barrier crossing relative to the center of the barrier at an imbibition width of 0, indicating the current DBD, which is reduced by an increase of droplet volume during the process; upon imbibition ring merging, no BaSO_4_ formation is observed at the contact line of the BaCl_2_ and MgSO_4_ solution imbibition rings.

To analyze barrier‐regulated imbibition, a first droplet was deposited on the MPS thin film surface directly next to the PMETA‐Cl barrier (NB or WB type). To ensure comparable preconditions in the liquid transport studies across the barriers, an identical droplet‐to‐barrier distance (DBD), measured between the droplet three‐phase contact line and the center of the barrier, was targeted (Figure 2). Since a DBD of a few hundred micrometers was found to be easily surmountable for all salt solutions, a DBD of 285 µm (±50 µm) was set for all experiments using a computer‐controlled droplet dispensing system. Further pursuing identical preconditions, the PMETA‐Cl barrier‐regulated imbibition experiments shown in Figure 2 were conducted at a constant temperature of 24 °C, a constant relative humidity of 50%, a constant salt concentration of 10 mmol L^−1^, and at fixed initial droplet volumes of 1.5 µL. These temperature, humidity, and concentration conditions were also maintained in droplet communication experiments, whereas droplet volumes were varied (Figure 3). The selected salt concentration ought to enable imbibition over a possibly large area while still allowing to screen salt‐specific electrostatic effects. According to the literature [22], this condition is met at salt concentrations of 10 mmol L^−1^, which allows for sustained imbibition advancement. Evaluating the potential of PMETA‐Cl barriers for ion permselective imbibition and ion retention, aqueous solutions of BaCl_2_, MgSO_4_, MgCl_2_, and NaCl were examined. These salts present different cations, varying in ionic charge, ionic radius, and hydration properties. Accordingly, differences in barrier permeation are expected due to ion‐specific interactions with the spatially confined polycationic PMETA + chains. The underlying mechanisms are likely governed by multiple physicochemical factors acting together, among which cation–cation repulsion within the confined polyelectrolyte regime is expected to contribute to reduced ion uptake [14]; all individual contributions are lumped into effective parameters and global resulting behaviors in the scope of the present work. Figure 2 shows a marked difference in the film imbibition regarding free‐ or barrier‐regulated imbibition. After some critical time, the wide barrier (WB) always acts as a physical barrier under the applied conditions, and liquid imbibition is arrested. In the case of a narrow barrier (NB), the imbibition is arrested for a short time period, followed by a breakthrough of the annulus that subsequently follows approximately a similar imbibition law.

In contrast to the examined salt solutions, which allow for fluid permeation through the NB (Figure 2a–d), complete barrier permeation of pure water could not be achieved even when using reduced DBDs relative to Figure 2a–d, which was ascribed to the lower imbibition widths and attenuated imbibition characteristic for pure water droplets (Figure S2) [22].

Comparing the time‐dependent imbibition profiles of Figure 2a–d shows that the initial imbibition and annulus formation (*t* < 5 s), as well as the advancing imbibition for *t* > 100 s of NB‐regulated and free imbibition, practically coincide. While the time‐dependent initial imbibition is well described by the evaporation‐modified Lucas–Washburn equation [20], the advancing imbibition at longer times, being reflected by continuously increasing imbibition widths, aligns with recent reports examining the ion‐specific imbibition behavior of aqueous salt solutions (*c* > 1 mmol L^−1^) into mesopores [21, 22]. In contrast to the steady‐state imbibition widths observed for water, where increased evaporation from the expanded fluid surface area halts further imbibition advancement, evaporation‐induced salt concentration effects rationalize the prolonged imbibition advancement of the studied salt solutions within the experimental timescale (Figure 2) [21, 22]. It is expected that the salt contents locally reduce the overall solvent evaporation rate, thus shifting the balance toward capillary forces, and enhance osmotic‐pressure‐driven fluid flows toward the imbibition zone, together counterbalancing the increased evaporation resulting from fluid surface area expansion and imbibition ring growth [21, 22].

At constant concentration, the barrier breakthrough time *t* *_a_* (red ellipses Figure 2a–d), which represents the duration between the first contact of the imbibition front with the visible barrier and its subsequent advancement at the opposite side beyond the barrier, decreases from 39.3 s for BaCl_2_ solution (Ba^2+^ radius in Ba(H_2_O)_8_^2+^ = 148 pm [45]), through 18.5 s for MgCl_2_ solution (Mg^2+^ radius in Mg(H_2_O)_6_^2+^ = 76 pm [45]) to 18.2 s for NaCl solution (Na^+^ radius in Na(H_2_O)_6_^+^ = 109 pm [45]) (Table 1). The salt‐specific trend of t_a_ in NB‐regulated imbibition was confirmed in repeated experiments, showing the averaged *t*_a_ to decrease according to the following order: BaCl_2_ > MgCl_2_ > NaCl (Figure S5). Comparing free and NB‐regulated imbibition shows that the NB does not significantly change the fluid imbibition kinetics itself, with the NB‐regulated and free imbibition processes showing equivalent dynamics. This conclusion is based on the cross‐correlation of NB‐regulated and free imbibition profiles (calculation described in the experimental section), describing the temporal relationship between both processes. Assigning values between 1 and −1, corresponding to a synchronous (+1), non‐correlated (0), and phase‐inverted (−1) behavior, the relationship of both imbibition profiles x(t) is evaluated (Table 1). The lag time describes whether x(t) evolves in phase or with a time shift between NB‐regulated and free imbibition. With consistent high positive cross correlations ≥0.89 at a lag time of 0 s, an almost synchronous relationship between the NB‐regulated and free imbibition profiles of BaCl_2_, MgSO_4_, NaCl, and MgCl_2_ without a delay is observed. Hence, analogous imbibition dynamics for NB‐regulated and free imbibition after barrier breakthrough are inferred, whereas a delay of the imbibition process due to the presence of the PMETA‐Cl barrier is not observed. The lower final imbibition ring widths of NB‐regulated imbibition as compared to free imbibition (Figure 2a–d) probably result from the time required for barrier breakthrough, delaying the overall imbibition advancement. To evaluate whether the imbibition widths of both processes diverge, the mean average deviation (MAD, calculation described in the experimental section) of imbibition widths between NB‐regulated and free imbibition was compared with the terminal deviation (TD) in imbibition widths between NB‐regulated and free imbibition at t_max_. In general, the measured TDs, with an average difference TD–MAD of approximately −10 µm (Table 1), were either lower than MAD or comparable to MAD, indicating that NB‐regulated and free imbibition of BaCl_2_, MgSO_4_, NaCl, and MgCl_2_ solutions evolve almost consistently. This observation is corroborated by the pronounced positive cross‐correlations of both processes (Table 1).

**TABLE 1:** DBD values, barrier breakthrough time (*t* *_a_* if applicable), and the comparison of barrier‐regulated (br‐reg.) against free imbibition (imb.) advancement x(t) through cross correlation at a lag time of 0 s as well as mean average deviation (MAD) (the calculation of cross correlation and MAD is described in the experimental section) and terminal deviation between both trends (for the terminal value of t).

**Exp**	Salt	Barrier	DBD (µm)	**Barrier Breakthrough Time (*t*** *_**a**_* **/s)**	**Cross‐Correlation (br‐reg./free imb.)** **{lag}**	MAD/Terminal Deviation (µm)
a)	BaCl_2_	NB	335.0	39.3	0.99 {0}	65/30
a)	BaCl_2_	WB	247.5	—	0.01 {0}	395/570
b)	MgSO_4_	NB	297.0	25.5	0.89 {0}	26/25
b)	MgSO_4_	WB	238.6	—	0.00 {0}	222/295
c)	NaCl	NB	262.4	18.2	0.96 {0}	24/14
c)	NaCl	WB	160.0	11.1	−0.30 {0}	147/305
d)	MgCl_2_	NB	250.0	18.5	0.98 {0}	60/67
d)	MgCl_2_	WB	255.0	—	0.00 {0}	328/464

When increasing the width of the PMETA‐Cl barrier (WB type of 114 ± 8 µm width on average, Figure S4a), the imbibition fronts of aqueous BaCl_2_, MgSO_4_, and MgCl_2_ solutions are arrested at the PMETA‐Cl barrier without passing it (Figure 2a–b,d), indicating aqueous salt solution retention by the barrier under the applied conditions. Only the imbibition front of the NaCl solution slightly extends across the WB without exhibiting net imbibition advancement over time upon barrier breakthrough (Figure 2d). In contrast, the free imbibition of NaCl solution continuously advances, which results in an increasing difference in imbibition width between WB‐regulated and free imbibition, as reflected by the terminal difference TD–MAD of + 158 µm combined with the low and negative cross‐correlation of −0.30. The facilitated permeation of the aqueous NaCl solution through the WB is ascribed to the specific properties of Na^+^, including its reduced charge and radius relative to Ba^2+^, as well as possible NaCl‐specific barrier interactions. Another potential aspect promoting the permeation of NaCl solution through the WB is the sub‐average DBD of the solution droplet (Table 1), enhancing fluid supply by increasing the solvent flow according to Darcy's law [5] and promoting vapor adsorption or condensation [46] into the PMETA‐Cl‐containing pores near the droplet. A potential reason for the absence of imbibition advancement of the NaCl solution after barrier breakthrough is ion retention by the barrier, which decreases the NaCl concentration beyond the barrier. The lower salt concentration is associated with weaker osmotically driven fluid pumping, impeding the overall imbibition advancement and possibly explaining the lack of further increase in imbibition ring width. Upon barrier breakthrough, the imbibition front beyond the barrier is observed to strongly oscillate (Figure 2d). This oscillation of the fluid front is in accordance with literature on imbibition of aqueous solutions without polymer barriers, in which the oscillation is ascribed to local water vapor capillary condensation into the silica mesopores and the hysteresis in the water vapor adsorption‐desorption isotherm [47].

Based on the different imbibition behaviors observed for the NB and WB, the PMETA‐Cl barrier width is identified as a key physical influence on barrier‐regulated aqueous salt solution imbibition. The comparison of NB‐ and WB‐regulated imbibition emphasizes the adaptable function of DLW‐fabricated PMETA‐Cl barriers, reaching from the ion‐specific regulation of solution flow (NB) to complete solution retention (WB). Molecular simulations [14, 48] and complementary spectroscopic techniques such as fluorescence lifetime imaging spectroscopy (FLIM) [49] and fluorescence correlation spectroscopy (FCS) promise further insights into the physical origins of the observed ion‐specific barrier permeation.

### 2.3 Enforcing Barrier Breakthrough and Ion Permeation

Increasing the droplet volume to 12 µL significantly prolonged the droplet evaporation times to more than 2000s and thus extended fluid–barrier interaction times, ultimately allowing for the barrier breakthrough of aqueous MgSO_4_ as well as BaCl_2_ solution through the WB (Figure 3). The droplet volumes were increased stepwise from an initial 1.5 to 12 µL while ensuring that the droplets neither extended across the barrier nor came into contact. The increased droplet volume naturally leads to a decrease in DBD by increasing the spatial extension of the droplet. Since low DBDs already appeared beneficial to the permeation of NaCl solution through the WB (Figure 2c, Table 1), the current DBDs were screened over the entire imbibition timescale in Figure 3 to examine the influence of DBD on fluid and ion transport across the barrier. The precipitation of BaSO_4_ upon contact of the 10 mmol L^−1^ MgSO_4_ and BaCl_2_ solution drop imbibition rings is used as a simple macroscopic readout of ion permeability according to previous literature [41]. Tracking the BaSO_4_ precipitation confirmed the transport of BaCl_2_ and MgSO_4_ solution across the NB (Figure S6, Figure S7), as already inferred from Figure 2a,b.

The WB‐regulated imbibition process of MgSO_4_ solution (Figure 3a) is subdivided in six characteristic steps: the imbibition barrier breakthrough after droplet deposition (step 1), the advancing imbibition at the opposite side of the WB (step 2), the steady‐state imbibition (step 3), the re‐initiated imbibition advancement (step 4), the imbibition upon deposition of the BaCl_2_ droplet at the opposite side of the barrier, resulting in imbibition ring merging (step 5), and BaSO_4_ precipitate formation at the contact line of both imbibition rings (step 6, Figure 3a).

In contact with the WB, the MgSO_4_ solution reaches a barrier breakthrough time t_a_ of 116.4 s (Figure 3a, step 1) as compared to a much shorter t_a_ of 25.5 s in contact with NB (Figure 2b), indicating an impeded ion permeation through the wider WB, which resulted in complete fluid retention during the lower imbibition times evaluated in Figure 2b. The 4.6‐fold increase of t_a_ for the WB relative to the NB, surpassing the 1.7‐fold increase in barrier width, indicates a nonlinear influence of the barrier width on t_a_. This is probably further related to the increased polymer amount usually observed for the higher laser powers used in the preparation of the broader WB type [28]. Up to *t* ≤ 450 s, a non‐advancing, slightly oscillating imbibition beyond the barrier predominates, representing an intermediate state between barrier breakthrough (step 1, Figure 3a) and imbibition advancement (step 2, Figure 3a). The non‐advancing imbibition implies a decreased ion concentration beyond the barrier, limiting the driving force for osmotic solvent pumping across the barrier. The resulting impeded imbibition advancement despite apparent barrier hydration identifies the barrier permeation by ions as slower than the barrier hydration alone. Although the droplet volume increases associated with decreased DBDs induce increased free imbibition rates (Figure S8), the gradual decrease of DBD during step 1 by 85 µm does not result in immediate imbibition advancement at the opposite site of the WB.

Only as the liquid–barrier interaction time progresses at constant DBD and droplet volume, the initially stagnant imbibition beyond the barrier (*t* < 450 s) transitions into continuously advancing imbibition up to *t* = 750 s (Figure 3a, step 2), showing that imbibition advancement develops over time. Notably, WB‐regulated (Figure 3a) and NB‐regulated (Figure 2b) imbibition of MgSO_4_ solution is observed to advance at an almost identical speed of 0.56 µm/s (WB) and 0.54 µm/s (NB) during the imbibition advancement phase (step 2), indicating similar imbibition dynamics as soon as the fluid front has crossed the barrier, despite the deviating barrier widths (NB: 49 µm/WB: 83 µm, method 1, Figure S4a) and DBDs (NB: 297 µm/WB: 474 µm). This unrestricted, advancing imbibition across the barrier demonstrates the absence of ion filtering by the barrier. The underlying unrestricted ion permeation through the polyelectrolyte barrier is considered crucial to the observed imbibition advancement beyond the barrier, driven by osmotic pumping.

Transitioning from step 2 to step 3 (Figure 3a), the decay of imbibition rates toward a steady state at *t* > 750 s indicates a decreasing influence of osmotic pumping in dependence on imbibition width. This apparent steady state (Figure 3a, step 3) is observed for the first time after increasing the imbibition time to *t* > 750 s. Interestingly, the steady‐state imbibition widths remain unaffected by several successive DBD decreases down to 262 µm, and the corresponding droplet volume increases to 10.5 µL, showing the relative independence of imbibition advancement from DBD and the droplet volume under these conditions. The observed steady state is related to equilibrating capillary imbibition and fluid evaporation as described by the evaporation‐modified Lucas–Washburn equation [20].

During step 4 (Figure 3a), the further decrease of DBD to a minimum of 169 µm at 980 s finally re‐initiates further imbibition ring growth and thus further increases imbibition widths with time, showing that lower DBDs can be used to shift the steady state toward higher imbibition widths. Notably, the re‐initiated imbibition requires a sufficiently low DBD of 169 µm, as corresponding to a droplet volume 12 µL, with a similar DBD promoting the accelerated barrier breakthrough of NaCl solution through the WB in Figure 2c. The re‐initiated imbibition (Figure 3a, step 4) and accelerated barrier breakthrough (Figure 2c) at low DBDs are ascribed to a higher salt solution flow according to Darcy's law [5] and potentially enhanced by pore condensation in proximity to the droplet three‐phase contact line [46].

In step 5 (Figure 3a), the final deposition of the BaCl_2_ solution as the second droplet on the opposite side of the barrier induces rapid merging of the MgSO_4_ and BaCl_2_ solution imbibition rings within 0.8 s under gradual formation of a visible line of solid precipitate at the contact line of both imbibition rings. This precipitate formation behind the barrier with respect to the first MgSO_4_ droplet clearly demonstrates the transport of SO_4_^2−^ ions through the barrier. The observed permeation of MgSO_4_ solution through the WB indicates that the retention of salt solution by the wider WB (Figure 2b) can be overcome by adjusting the fluid–barrier interaction times. The observed rapid convergence of the MgSO_4_ and BaCl_2_ solution annuli upon the deposition of the BaCl_2_ droplet within 0.8 s may be supported by enhanced capillary condensation in the inter‐droplet spacing. Capillary condensation into mesopores is reported for increased relative humidities > 60% [46, 50], which may be locally exceeded in the inter‐droplet spacing due to solvent evaporation.

All these observations indicate the relevance not only of electrostatic interaction but also of the relative interplay of polymer amount, barrier width, and barrier position with respect to the droplet and within the imbibition ring.

When looking at the BaCl_2_ solution in NB‐regulated imbibition (Figure 2a, Figure S5), higher t_a_ values indicating an inhibited imbibition relative to MgSO_4_ (Figure 2b, Figure S5) were already observed. To evaluate whether the WB nevertheless allows for permeation of BaCl_2_ solution, the procedure of Figure 3a was applied, inverting the order of droplet deposition to first BaCl_2_ followed by the deposition of a MgSO_4_ solution droplet on the other side of the PMETA‐Cl barrier (Figure 3b). In contrast to the WB‐regulated imbibition of MgSO_4_ solution (Figure 3a), the WB‐regulated imbibition of BaCl_2_ solution does not involve an advancing imbibition phase upon barrier breakthrough (steps 1–2, Figure 3b). The imbibition front even retracts (step 3), whereas a strong advance is only induced by the final deposition of the second MgSO_4_ droplet at the opposite side of the barrier (step 4). Thus, the process starting with the initial deposition of BaCl_2_ solution is subdivided into the following characteristic steps: the imbibition barrier breakthrough (step 1), the oscillating imbibition (step 2), the retracting imbibition (step 3), and the imbibition following the deposition of the MgSO_4_ droplet, resulting in imbibition ring merging (step 4, Figure 3b).

Analyzing step 1 (Figure 3b), the barrier breakthrough time *t* *_a_* of BaCl_2_ solution through the WB is significantly increased to 347 s as compared to the smaller NB, showing a breakthrough time of 39 s (Figure 2a). This almost nine‐fold increase in *t* *_a_* relative to a threefold increase in barrier widths clearly overcomes the 4.6‐fold increase observed for MgSO_4_ solution (Figure 3a), showing the relatively increased resistance of the PMETA‐Cl barrier against BaCl_2_ solution permeation in line with previous results (Figure 2a,b, Table 1, Figure S4a). The impeded imbibition of the BaCl_2_ solution through the WB further substantiates the slower permeation behavior of Ba^2+^ compared to Mg^2+^ for PMETA‐Cl barrier permeation inferred from Figure 2, which can be ascribed to the larger ionic radius of Ba^2+^.

In step 2 (Figure 3b), the imbibition advancement of BaCl_2_ solution, with an overall rate of 0.12 µm/s between 410 and 910 s remains significantly slower as compared to the 3.3‐fold increased imbibition speed of MgSO_4_ solution under equivalent conditions (Figure 3b), while underlying strong oscillations. The relatively reduced imbibition rates of BaCl_2_ solution identify the PMETA‐Cl barrier as responsible for the observed imbibition speed reduction, as the free imbibition of the BaCl_2_ solution is faster than the free imbibition of the MgSO_4_ solution (Figure 2a,b). External effects are considered negligible, as the ambient experimental conditions were controlled and constant over time, ensuring consistent solvent evaporation and condensation for the experiments. The simultaneously observed pronounced oscillations of imbibition width are characterized by a MAD from the 50‐point moving average of 28 µm for BaCl_2_ solution (Figure 3b) against 11 µm for MgSO_4_ solution (Figure 3a). This oscillating WB‐regulated imbibition of BaCl_2_ solution, including alternating expansion and retraction of the imbibition front, resembles WB‐regulated imbibition of NaCl solution (Figure 2c) while being absent in other salt solutions. The related complex imbibition dynamics are ascribed to the BaCl_2_ concentration differences in the imbibition zones on the droplet side of the barrier and beyond the barrier. Ion retention by the PMETA‐Cl barrier is associated with a higher ion concentration on the first droplet side of the barrier than in the imbibition zone beyond the barrier. Thus, facilitated solvent evaporation in the imbibition zone beyond the barrier may cause the significant imbibition front retractions observed (Figure 3b, step 2). Contrarily, an adverse solvent re‐supply across the barrier induced by solvent depletion beyond the barrier and the related concentration increase of remaining BaCl_2_ may explain the subsequent imbibition front expansions (Figure 3b, step 2). The lower BaCl_2_ concentration in the imbibition zone beyond the barrier is associated with a reduced osmotic pressure, ultimately resulting in the suppressed imbibition advancement of BaCl_2_ solution (Figure 3b, step 2) as contrasted with the unrestricted imbibition advancement of MgSO_4_ (Figure 3a, step 2). Consequently, the ion filtering by the PMETA‐Cl barrier, causing ion retention, is viewed as the key factor preventing osmotically driven imbibition advancement of BaCl_2_ solution beyond the barrier, as observed for MgSO_4_ solution. In step 3 (Figure 3b), the increase in imbibition width to a maximum of 234 µm at 976 s following the reduction of DBD to 315 µm, corresponding to a droplet volume increase to 12 µL, is found to be transient and is superseded by a retraction of the imbibition front back to the barrier, emphasizing the stagnating imbibition of BaCl_2_ solution over time. Under these conditions, an insufficient driving force for sustainable imbibition advancement, as observed for MgSO_4_ solution at further reduced DBDs (Figure 3a, step 4), is assumed.

In step 4 (Figure 3b), the final deposition of the MgSO_4_ solution droplet beyond the barrier does not lead to the formation of a consistent line of BaSO_4_ precipitate upon imbibition annuli contact and up to the increased time of 2300s, indicating substantial Ba^2+^ retention by the WB, preventing visible precipitate formation. The comparison to Figure 3a, where visible precipitation occurs, illustrates the distinct and ion‐specific WB‐regulated imbibition behavior of BaCl_2_ and MgSO_4_ solutions, underlying ion filtering by the barrier and unrestricted barrier permeation, respectively. Furthermore, the fundamentally different imbibition sequences of BaCl_2_ and MgSO_4_ solutions (Figure 3) upon barrier breakthrough emphasize the deviant imbibition behavior in both cases. The sustainable permeation of BaCl_2_ in contrast to MgSO_4_ solution through the WB could not be enforced by adjusting experimental parameters such as DBD and fluid–barrier interaction times. With PMETA‐Cl barriers, we presented a macromolecular device actively discriminating against the permeation of larger ions such as Ba^2+^ under nanoconfinement while enabling the permeation of smaller ions such as Mg^2+^, within the range of cations investigated.

## 3 Conclusions

We have introduced polyelectrolyte barriers to control fluid and ion transport along nanoporous matrices that act as droplet‐to‐droplet communication soft devices across a surface. Specifically, DLW‐fabricated linear PMETA‐Cl polymer lines in MPS thin films served as ion‐specific fluid gating control in barrier‐regulated imbibition experiments using aqueous salt solution droplet compartments. The ion‐specific permeation behavior was quantified by barrier‐breakthrough times (*t* *_a_*), which were found to increase in the order Na^+^ > Mg^2+^ > Ba^2+^. The solution permeability of the PMETA‐Cl barriers proved to be tunable by adjusting the barrier width from 33 µm to 125 µm along with polyelectrolyte densities, allowing control from normal permeation (narrower barriers) to retention (wider barriers) of BaCl_2_, MgSO_4_, MgCl_2_, and NaCl solutions under the applied conditions. Ion‐specific interactions with the barrier led to distinct imbibition dynamics. Depending on the ion type and DBD, imbibition can either proceed beyond the barrier toward a steady state, display oscillatory dynamics near the outer rim of the barrier, or become effectively suppressed. These behaviors reflect different transport regimes, ranging from unrestricted permeation to partial filtering and, ultimately, ion exclusion by the barrier. Unrestricted transport was particularly observed for narrower PMETA‐Cl barriers, prolonged fluid–barrier interaction times, smaller cation types such as Mg^2+^, and cation types with lower charges such as Na^+^. Partial ion retention at the barrier was typical for wider barriers and larger cation types such as Ba^2+^. Complete retention was ensured by wider barriers and short fluid–barrier interaction times. A characteristic indicator for ion filtering by the barrier was strongly oscillating imbibition beyond the barrier, as observed for NaCl and BaCl_2_ solutions. The ion‐selective barrier permeability and gating function were also optically revealed by using a precipitation reaction as readout. The crossing of MgSO_4_ solution through the barrier, in contrast to the retention of BaCl_2_ ions by the barrier under equivalent conditions, was corroborated by the presence/absence of BaSO_4_ precipitate formation at the contact line of the MgSO_4_ and BaCl_2_ solution annuli in droplet communication. The results show that the chemical communication between two distinct droplet compartments in nanofluidic contact through a mesoporous thin matrix can be regulated by submillimeter‐scale polyelectrolyte barriers. Together, these findings open a new avenue for the rational design of intelligent micro/nano fluidic circuits, with potential applications in digital microfluidics, on‐chip‐based sensing, and, as a longer‐term perspective, the emulation of cell‐like chemical communication.

## 4 Experimental Section

### 4.1 Materials and Procedures

Sodium diethyldithiocarbamate trihydrate, 4‐(chloromethyl)phenyltrimethoxysilane, tetraethyl orthosilicate (TEOS, 98%), Pluronic F127 (BioReagent grade, 12.6 kg/mol), absolute ethanol (EMPLURA, 99.5%), hydrochloric acid (HCl, 37% in water, ACS reagent grade), dimethyl sulfoxide (DMSO, EMPLURA, 99%), [2‐(methacryloyloxy)ethyl]trimethylammonium chloride (METAC) solution in water (75%, stabilized with 4‐methoxyphenol), (5,10,15,20‐Tetraphenyl‐*21H*,*23H*‐porphine zinc) (ZnTPP), barium chloride (anhydrous, 99.99%, trace metals basis), magnesium sulfate (anhydrous, 99.5%, Reagent Plus), sodium chloride (99%, ACS reagent), and magnesium chloride (98%) were purchased from Sigma‐Aldrich (St. Louis, MO, USA), Merck (Darmstadt, Germany), or TCI (Tokyo, Japan) and used without further purification unless otherwise noted. Alexa Fluor 488 (A30629) was purchased from ThermoFisher Scientific (Waltham, MA, USA). Deuterated chloroform (CDCl_3_, 99.8% atom% D) was purchased from Sigma‐Aldrich. Anhydrous tetrahydrofuran (99.9%, anhydrous, containing 250 ppm BHT as inhibitor) and anhydrous toluene (99.8%) were purchased from Sigma‐Aldrich. Unless otherwise noted, deionized water was used. Solvents not mentioned above were technical grade. Reactions sensitive toward air exposure were carried out under inert conditions, using standard Schlenk line techniques under a nitrogen atmosphere (5.0, 99.999% purity, Linde, Dublin, Ireland).

CAs were measured as deposited with an OCA 35 device by DataPhysics using the SCA 4.5.2 software and the sessile droplet method under standard atmosphere (T = 23 °C, RH = 60%). A droplet volume of 2 µL was used, and the CA value was obtained by fitting the droplet shape using the approximation algorithm of the SCA software. The mean CA was measured for at least five droplets.

**Ellipsometry** was employed for determination of refractive indices and thicknesses of MPS thin films deposited on silicon wafer substrates (Si‐Mat, Kaufering, Germany, 100 mm diameter, 525 ± 25 µm thickness, type P/Bor, <100> orientation, CZ growth method, 2–5 W resistivity, polished on one side) using the nanofilm EP3−SE device (Accurion, Göttingen, Germany) equipped with a 658 nm laser. The software EP4‐View and EP4‐model (version 1.2.0) were used for measurements and model analysis. The angle of incidence (AOI) was varied from 38° to 68° in 2° increments and measured in one‐zone mode (Si wafer → SiO_2_ oxide layer → SiO_2_ mesoporous layer). The measurements were performed at 15% relative humidity, adjusted by the MHG32 Modular Humidity Generator (ProUmid, Ulm, Germany). The refractive index (n) and layer thickness (d) of the samples were fitted through the EP4 software in up to 1000 iterations, approaching a terminal error tolerance of 10^−7^. The fits were performed within the ranges 1.0 ≤ *n* ≤ 1.5 and 500 nm ≤ *d* ≤ 700 nm. The calculation of porosity was carried out using the obtained average refractive indices of n = 1.228 ± 0.01 for MPS thin films (refractive index of dense silica = 1.46) [51] according to Brüggemann's effective medium theory described in the literature [52]. The film porosity was measured at the center of the film at three different positions for three independent thin films.

**Transmission Electron Microscopy** (TEM) was performed on a Philips FEI CM20 (FEI Philips Electron Optics, Hillsboro, OR, USA) microscope equipped with a LAB‐6 cathode and an Olympus CCD camera (Olympus, Tokyo, Japan). The acceleration voltage was set to 200 kV for operation. MPS thin films were scratched off the substrate and suspended in ethanol using an ultrasonic bath. A droplet of the ethanolic suspension was placed on a holey carbon‐coated copper grid before the measurement. The bright pore voids in the respective TEM images were modeled as ellipses. The pore diameter was determined as the Feret diameter of the modeled ellipses using the ImageJ software by Fiji [53]. The reported values correspond to the pore diameters at the neck.

**NMR spectra** were recorded on a Bruker AC 300 (Bruker, Billerica, MA, USA) and analyzed using the software MestreNova 14.2.3. Chemical shifts (*δ*) are reported relative to trimethylsilane (TMS) and calibrated on the residual peak of deuterated solvents (CDCl_3_ *δ* = 7.26) [54]. Coupling constants (*J*) are given in Hz. The following abbreviations were used for signal multiplicities: singlet (s), doublet (d), triplet (t), quartet (q), quintet (p), multiplet (m), broad (br), and combinations thereof.

**Light intensity measurements** were performed using a PM160T Optical Power Meter (ThorLabs, Newton, NJ, USA) with a detector area of 0.71 cm^2^. Light intensities were determined at 405 nm 10 s after switching on the light source. During the measurement, the detector was mounted at a fixed distance from the light source.

**SBDC (4‐(trimethoxysilyl)benzyl diethylcarbamodithioate)** was synthesized according to the literature [55]. Sodium diethyldithiocarbamate trihydrate was dissolved in hot methanol and filtered while hot to remove the insoluble residue. Then the solvent was removed under reduced pressure, and the so‐obtained purified sodium diethyldithiocarbamate was dried at 0.05 mbar overnight. Under an inert gas atmosphere, purified sodium diethyldithiocarbamate (3.08 g, 18.0 mmol, 1.00 eq.) was dissolved in anhydrous THF (20 mL). In a second flask 4‐(chloromethyl)phenyltrimethoxysilane (4.02 mL, 4.50 g, 18.2 mmol, 1.01 eq.) was dissolved in anhydrous THF under an inert gas atmosphere. Under stirring, the solution of sodium diethyldithiocarbamate was added dropwise to the solution of 4‐(chloromethyl)phenyltrimethoxysilane. The mixture was stirred for 4.5 h at room temperature. Subsequently, the formed solid residue was filtered out. The solvent was removed under reduced pressure. The so‐obtained milky, yellowish oil was dried overnight at 0.05 mbar. Its NMR spectrum complies with the literature [55]: ^1^H NMR (300 MHz, CDCl_3_, δ): 7.59 (d, *J* = 8.3 Hz, 2H, Ar‐H), 7.41 (d, *J* = 8.3 Hz, 2H, Ar‐H), 4.55 (s, 2H, CH_2_—S), 4.04 (q, *J* = 6.6 Hz, 2H N—CH_2_—CH_3_), 3.72 (q, J = 6.6 Hz, 2H, N—CH_2_—CH_3_), 3.61 (s, 9H, O—CH_3_), 1.28 (t, J = 6.6 Hz, 6H, N—CH_2_—CH_3_) ppm.

**Mesoporous silica thin films** were prepared through sol–gel chemistry using tetraethyl orthosilicate (TEOS) as an inorganic precursor and the amphiphilic triblock copolymer Pluronic F127 as a structure‐directing template that undergoes micellization upon solvent evaporation. According to reported protocols [56], the molar ratios of the sol–gel precursor solutions were set to 1 TEOS: 0.008 Pluronic F127: 16.4 ethanol: 14 water: 0.015 HCl. According to the molar ratios, the following quantities were used: TEOS (9.76 mL, 10.4 g, 50.0 mmol), Pluronic F127 (5.22 g, 0.41 mmol), ethanol (48.0 mL, 37.9 g, 822 mmol), water (6.40 mL, 6.39 g, 354 mmol), aqueous hydrochloric acid (6.40 mL, 0.10 mol/L). The preparation of the sol–gel precursors was performed at room temperature, first suspending Pluronic F127 in ethanol under stirring. Upon addition of a freshly prepared solution of hydrochloric acid (37%) in water (0.05 mol L^−^^1^), the template dissolved immediately. After the addition of TEOS, the solution was stirred overnight at room temperature and subsequently stored at −18 °C. Before dip coating, the sol–gel solutions were stirred for 30 min at room temperature, and the silicon wafer substrates (Si‐Mat, Kaufering, Germany, 525 ± 25 µm thickness, type P/Bor, <100> orientation, CZ growth method, 2–5 W resistivity, polished on one side, 7 × 2 cm size) were cleaned with ethanol‐soaked fabrics. Then, the substrates were dip‐coated at a withdrawal speed of 2 mm s^−1^ under controlled environmental conditions (51% relative humidity, 23 °C) using the previously reported EISA process [42]. After aging under the controlled climate conditions for 60 min, the films were subjected to the following temperature treatment: heating up to 60 °C within 10 min and holding the temperature for 60 min, followed by a temperature increase to 130 °C within 10 min. and holding the temperature for 60 min. Subsequently, the temperature was increased to 350 °C with a heating rate of 1 K min^−1^, maintaining the final temperature for 2 h.

**Functionalization of mesoporous silica thin films with SBDC.** After charging two Schlenk tubes with 18 samples (MPS thin films deposited on silicon wafer substrates), a solution of the SBDC (91.0 mg, 253 µmol) in anhydrous toluene (110 mL) was added under an inert gas atmosphere. The reaction vessels were heated for 1 h at 80 °C in the dark. After cooling down, the substrates were extracted in toluene for 10 min, rinsed with ethanol, and dried in air. The so‐obtained SBDC functionalized MPS thin films were analyzed through CA measurements.

**ZnTPP catalyzed laser writing in MPS thin films.** In a 50 mL screw‐lid jar, a solution of METAC (75% in water, 7.67 g, 14.7 mmol, 1.00 eq.) and ZnTPP (1.00 mg, 1.47 µmol, 1.00 eq. 10–4 eq.) in DMSO (10 mL) was prepared and subjected to an ultrasonic bath for 3 minutes to complete the dissolution of ZnTPP. After covering the aperture of a 60x objective with immersion oil (Type F, Nikon, Tokyo, Japan), a glass Petri dish was placed above the objective on the sample platform of a Nikon Eclipse TI2‐E laser microscope (Nikon, Tokyo, Japan). After establishing contact between the objective and the Petri dish, the glass container was filled with the polymerization solution. A silicon wafer substrate coated with a MPS thin film and functionalized with SBDC was immersed in the polymerization solution and placed face‐down on two 2 mm high PTFE plates positioned at both ends of the Petri dish so that a gap between the substrate and the bottom of the Petri dish was formed. To remain in position throughout the DLW process, the substrate was weighed above the PTFE plates using 50 g iron cuboids. After incubation in the polymerization solution for 10 min, DLW was performed. Under protection from external light, an array of 4000 positions was irradiated with 405 nm laser under controlled irradiation times (1 s per position) and powers (15 mW initial laser power for the NB type, and 25 mW as well as 33 mW for the WB type (the individual barrier widths are shown in Figure S4, the denoted laser powers correspond to the output laser powers in Figure S9)) using a beam expander setting of “8x.” The targeted arrays of positions were encoded in a list of x/y coordinates using the built‐in macro function of the operating software, which directed the motorized sample stage while opening and closing the microscope's shutter. To generate a coherent polymer barrier, an interposition distance of 10 µm was selected. Upon irradiation, the substrates were rinsed with ethanol and water, and then extracted in water for 30 min, in dilute hydrochloric acid (pH 3) for 30 min, and in ethanol for 10 min. The samples were dried in air.

**Droplet imbibition experiments.** For performing droplet imbibition experiments with aqueous salt solutions of 1) BaCl_2_, 2) MgSO_4_, 3) MgCl_2_, and 4) NaCl, characterized by a concentration of 10 mmol L^−1^, the respective salts were dissolved in MilliQ water using the following quantities: 1) 101.2 mg of BaCl_2_ in 48.6 mL of water, 2) 57.8 mg of MgSO_4_ in 48.0 mL water, 3) 57.8 mg of MgCl_2_ in 50.0 mL water, and 29.2 mg of NaCl in 50.0 mL water. Droplet imbibition experiments were conducted under a standard atmosphere (*T* = 24 °C, relative humidity = 50%) on SBDC‐functionalized MPS thin films deposited on reflective silicon wafer substrates with implemented PMETA‐Cl barriers. Aqueous salt solution droplets of 1.5 µL volume were deposited on the substrate surface at a predefined droplet barrier distance (DBD) using a computer‐controlled droplet dispensing system of an OCA35 instrument (DataPhysics, Filderstadt, Germany). The entire liquid imbibition process was recorded at a frame rate of 11 s^−1^ using the Topview Video System TVS‐C (software SCA 4.5.2 Build 1052, DataPhysics, Filderstadt, Germany) until full evaporation of all involved droplets. The videos were recorded at a zoom of 1.0x and a fixed brightness. The droplet volume was expanded from an initial volume of 1.5 µL in steps of 1.5 µL using either the computer‐controlled droplet dispensing system or an Eppendorf pipette. After performing and evaluating the imbibition experiments, the samples were analyzed through fluorophore adsorption followed by fluorescence microscopy to confirm the formation of PMETA‐Cl barriers while avoiding the interference of the fluorophore species with the imbibition process.

**The imbibition widths** were determined from the video footage using the software Tracker 6.3.0 by Open Source Physics based on the refractive index changes at the imbibition front. Within the first 2 s of the imbibition process, the imbibition widths were determined for every frame, then in 2 s steps until the commencement of droplet deformation due to advancing solvent evaporation. Imbibition widths were measured in a predefined direction between the droplet three‐phase contact line and the imbibition front. In barrier‐regulated imbibition experiments, the imbibition width advancement was determined in the perpendicular direction to the PMETA‐Cl barrier. So‐obtained imbibition widths in pixel units were transformed into µm based on the reference scale, recorded prior to each imbibition experiment. The imbibition widths in Figure 3 are referenced to the center of the barrier (imbibition width of 0), only showing the imbibition process beyond the barrier at imbibition widths ≥ 0.

**The droplet barrier distances (DBDs)** were determined from the video footage using the ImageJ software by Fiji [53]. DBDs were measured in a perpendicular direction to the barrier, between the droplet three‐phase contact line and the center of the barrier. So‐obtained imbibition widths in pixel units were transformed into µm based on the reference scale, recorded prior to each imbibition experiment.

**Visible PMETA‐Cl barrier widths** were determined from the video footage collected during the imbibition experiments. Initially, the orthogonal brightness profiles of each barrier section, participating in individual imbibition experiments, were determined at five significant positions along the barrier using the ImageJ software by Fiji [53]. The so‐obtained brightness profiles relate GVs to a length scale whose initial unit was transformed into µm based on the reference scale. Unless otherwise noted, the brightness profiles were measured prior to drop deposition. For computerized determination of barrier widths the collected GV profiles with the length scale x were modeled through Gaussian functions by using Origin2023 Pro (Orthogonal Distance Regression, method 1) according to Equation (1):

(1) $$\text{GV}\left(x\right)=GV_{0}+\left(\frac{A}{w\sqrt{\frac{\pi }{2}}}\right)\;e^{-2{\left(\frac{x-x_{c}}{w}\right)}^{2}}$$

The so‐obtained functions were resolved according to the following condition (Equation (2)):

(2) $$\text{GV}\left(x\right)=GV_{0}+\frac{A}{2w\sqrt{\frac{\pi }{2}}}$$

The following result was obtained (Equation (3)):

(3) $$x_{1}=x_{c}+w\cdot \sqrt{\frac{\ln\;\left(2\right)}{2}};\;x_{2}=x_{c}-w\cdot \sqrt{\frac{\text{ln}\;\left(2\right)}{2}}$$

The width b at FWHM was defined according to Equation (4):

(4) $$b=\left|x_{1}-x_{2}\right|=2w\cdot \sqrt{\frac{\text{ln}\;\left(2\right)}{2}}$$

The barrier widths for all barrier sections were calculated according to Equation (4) and averaged. Due to the low contrast between the barrier and the surrounding film surface, several GV profiles required additional smoothing to enable meaningful Gauss modeling (indicated in Figure S4). For an extended examination, the GV profiles obtained from WB2 (Figure 3) were additionally modeled through an alternative procedure (method 2) involving the Gaussian function in Equation (5):

(5) $$\text{GV}\left(x\right)=GV_{0}+Ce^{-\frac{1}{2}{\left(\frac{x-x_{c}}{w}\right)}^{2}}$$

During the modeling process, the amplitude C was fixed to the maximum of the respective GV profile. The so‐obtained functions were resolved according to the following condition (Equation (6)):

(6) $$GV\left(x\right)=GV_{0}+\frac{C}{2}$$

At FWHM, the following width was determined (Equation (7)):

(7) $$b=\left|x_{1}-x_{2}\right|=2w\cdot \sqrt{2\ln\;\left(2\right)}$$

The barrier widths obtained from Equation (7) were averaged. The calculated barrier widths are shown in Figure S4.

**The cross‐correlation of two data series at different lag times.** To analyze the imbibition processes, imbibition widths *x* were recorded as a function of time *t*. The comparison of imbibition processes through cross‐correlation comprised two data series corresponding to the same salt solution and experiment. In general, barrier‐regulated imbibition *x*_1_(*t*), and free imbibition *x*_2_(*t*) were compared. The comparison was performed within a predefined time frame with the limits *t*_0_ (time of barrier breakthrough) and *t*_max_ (time of evaporation‐related droplet deformation). First, the means of both data series *µ*_1_ and *µ*_2_ were calculated. Then, the standard deviations of both data series *σ*_1_ and *σ*_2_, as well as the number of compared data points N, were calculated. The cross‐correlation was obtained by calculating the normalized Pearson correlation coefficient [57] *ρ*_12_ (Equation (8)) for the integer lag times *t*_lag_, reaching from −10 to 10 s.

(8) $$\rho_{12}=\frac{\sum_{t=t_{0},\Delta t=2s}^{t_{\text{max}}}\left(x_{1}\left(t\right)-\mu_{1}\right)\left(x_{2}\left(t\right)-\mu_{2}\right)}{N\cdot \sigma_{1}\cdot \sigma_{2}}$$

At negative lag times, the data series *x*_2_(*t*) was shifted relative to *x*_1_(*t*), with *x*_1_(*t*) kept static, by the absolute value of the lag time. At positive lag times, *x*_1_(*t*) was shifted relative to *x*_2_(*t*), with *x*_2_(*t*) kept static. At a lag time of 0, no shifting was applied.

**Calculation of the mean average deviation (MAD).** To analyze the imbibition processes, imbibition widths *x* were recorded as a function of time *t*. The comparison of imbibition processes through MAD comprised two data series corresponding to the same salt solution and experiment. In general, barrier‐regulated imbibition *x*_1_(*t*), and free imbibition *x*_2_(*t*) were compared. The comparison was performed within a predefined time frame with the limits *t*_0_ (time of barrier breakthrough) and *t*_max_ (time of evaporation‐related droplet deformation). MAD was calculated according to Equation (9), with the number of compared data points N:

(9) $$\text{MAD}=\frac{\sum_{t=t_{0},\Delta t=2s}^{t_{\text{m ax}}}|\;x_{1}\left(t\right)-x_{2}\left(t\right)|}{N}$$

**PMETA‐Cl barrier imaging upon fluorophore adsorption.** After performing and evaluating the imbibition experiments, the PMETA‐Cl functionalized samples were incubated in a solution of Alexa 488 in water (1 µg mL^−1^) for 10 min according to a reported procedure [28, 40]. Then, the samples were extracted for 30 min in water and subsequently subjected to fluorescence microscopy. The related microscopy images were recorded by epi‐fluorescence imaging, using a fluorescent lamp (SOLA Light Engine, Lumencor, Beaverton, OR, USA) as part of the TI2‐E microscopy setup for widefield irradiation of the samples at an excitation wavelength of 488 nm. The images were recorded using an Andor ZYLA 4.2 PLUS camera (Andor Technologies, Belfast, UK) while tracking the emission wavelength in the range of 500–550 nm (Chroma Laser Filter set F46‐103, AHF analysentechnik AG, Tübingen, Germany).

## Funding

This study was supported by Deutsche Forschungsgemeinschaft (AN1301/4 and AN1301/8), European Research Council (803758), Consejo Nacional de Investigaciones Científicas y Técnicas (PhD scholarship), AGENCIA (CONVE‐2023‐100389751‐APN‐MCT), and AFOSR (FA9550‐24‐1‐0209).

## Conflict of Interest

The authors declare no conflicts of interest.

## Supporting information

Supplementary Material

## Data Availability

The data that support the findings of this study are available in the supplementary material of this article.
