# Supplementary material for: Programmable Inter‐Droplet Communication by Polymer‐Gated Ion Transport Through Nanopore Lattices
Source: Small Sci. 2026 Jul 29;6(8):e70352. doi: 10.1002/smsc.70352 (PMC13431961; doi:10.1002/smsc.70352)
Supplement: Supplementary file 1 — Supplementary Material [file SMSC-6-e70352-s001.pdf]

Supporting Information

Programmable Inter-Droplet Communication by Polymer-Gated Ion Transport through Nanopore Lattices

Marius Kirsch[#], Agustin D. Pizarro[#], Martín G. Bellino, Galo J. A. A. Soler-Illia*, and Annette Andrieu-Brunsen**

[#] Marius Kirsch and Agustin D. Pizarro contributed equally to this work and share the first authorship.

M. Kirsch, A. Andrieu-Brunsen
Ernst-Berl Institut für Technische und Makromolekulare Chemie, Technical University Darmstadt, Peter-Grünberg-Straße 8, 64287 Darmstadt, Germany
E-Mail: annette.andrieu-brunsen@tu-darmstadt.de

A. D. Pizarro, G. J. A. A. Soler-Illia
Instituto de Nanosistemas, Escuela de Bio y Nanotecnologías, (INS-EByN-UNSAM-CONICET), Av. 25 de Mayo 1169, 1650 San Martín, Argentina.
E-Mail: gsoler-illia@unsam.edu.ar

M. G. Bellino
Instituto de Nanociencia y Nanotecnología (CNEA-CONICET), Av. Gral. Paz 1499, San Martín, Buenos Aires, Argentina.
E-Mail: mgbellino@conicet.gov.ar

1. Transmission Electron Microscopy of MPS Thin Films

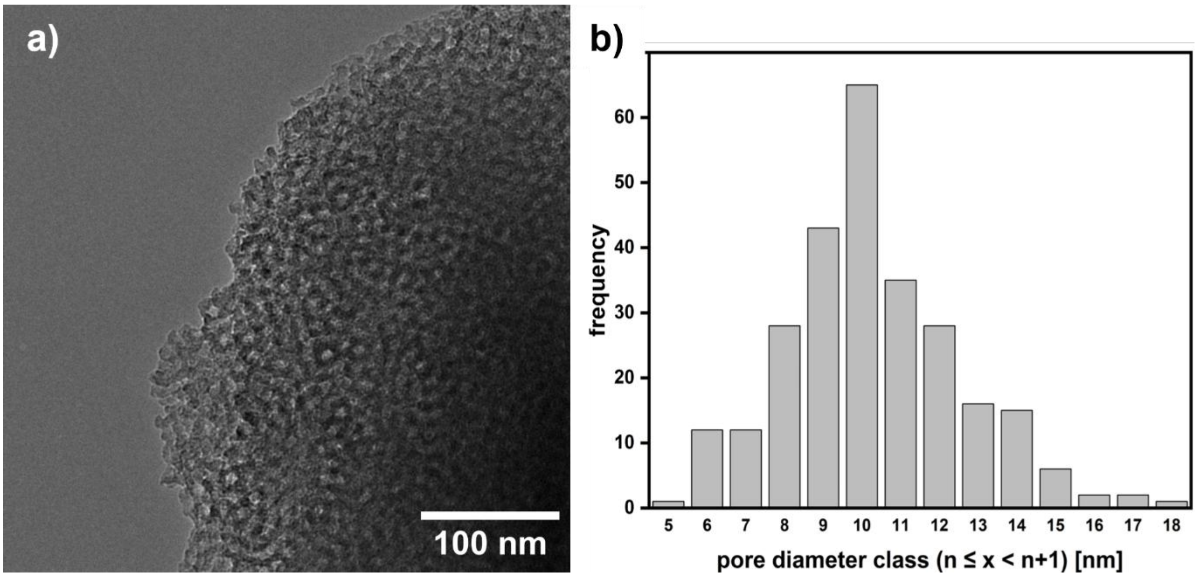

Figure S1. a) Transmission electron micrograph of the unfunctionalized mesoporous silica thin film; b) pore diameter distributions determined from a), with the two most abundant pore diameters highlighted (size ranges ascending from left to right in steps of 1 nm.

To measure the pore diameters characteristic for the MPS thin films studied, an exemplary MPS thin films was subjected to transmission electron microscopy (TEM) before iniferter and polymer functionalization. The so-obtained TEM image Figure S1a shows a pore size distribution ranging from diameters of 5 nm to diameters of 20 nm, with the most abundant pore diameters 10-11 nm (Figure S1b) and an average pore diameter of 11 ± 2 nm (determination of pore sizes is described in the experimental section). Similar pore size distributions are characteristic for MPS thin films prepared by Evaporation-Induced Self-Assembly-mediated dip coating.[1,2]

2. Imbibition of Water in MPS Thin Films

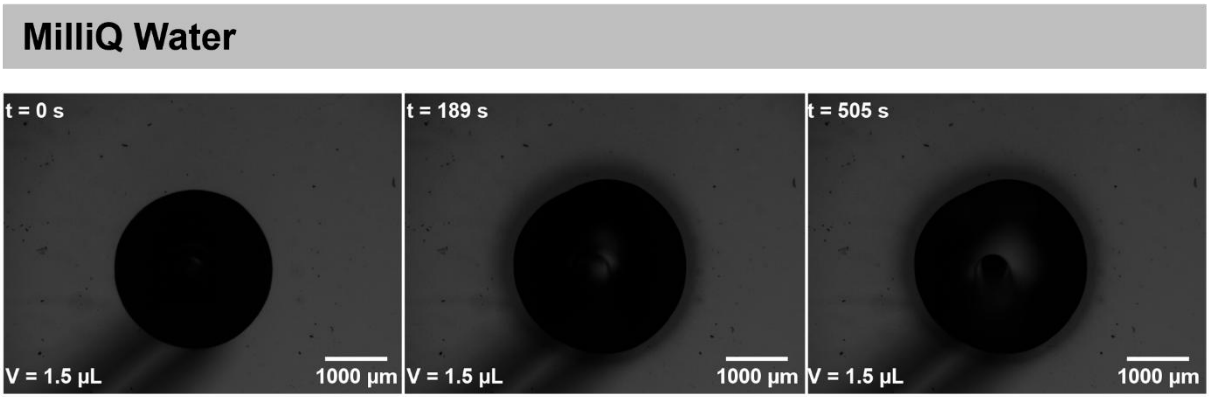

Figure S2. Temporal evolution of water imbibition in an SBDC-functionalized MPS thin film without barrier.

To demonstrate the suitability of SBDC-functionalized MPS thin films for water imbibition and examine the characteristic imbibition behavior of water, a droplet of water was deposited on a SBDC-functionalized MPS thin film without polymer barrier. Figure S2 shows the imbibition of water in dependence of time, clearly confirming the suitability of the material for water imbibition. 189 s after deposition of the water droplet an averaged imbibition width 206.7 ± 19.3 µm is measured. Up to times > 500 s no substantial imbibition advancement is measured, indicating the steady state dynamics typical for water.[3] Due to the absence of salt, no osmotic pumping is possible which significantly limits the attainable imbibition width according to the evaporation-modified Lucas-Washburn equation.[4] In comparison to the salt solutions examined, the maximum imbibition widths of water at t = 189 s are more than 50% decreased.

3. DLW-Mediated Post Grafting and Fluorophore Colonization of PMETA-Cl Polymer Barriers

To incorporate PMETA-Cl polymer barriers into the MPS thin films, DLW-mediated polymer post grafting was applied, a method developed in our previous works.[5,6] The method is based on PET-RAFT polymerization involving a the silica-bound photoiniferter SBDC, the dissolved photocatalyst ZnTPP, and the monomer META-Cl, polymerizing to silica-bound PMETA-Cl (chemical structures of the reactants in Figure S3a). The polymer formation is limited to the narrow laser light beam, thereby precluding polymer formation in regions that were not illuminated (Figure S3b).[5] Fluorophore colorization of the polyelectrolyte upon extraction highlighted the regular and consistent linear shapes of the polyelectrolyte barriers (Figure S3c-d). The different widths of the NBs and WBs resulted from varying the laser powers during DLW (experimental section). The increased width of the barriers observed in fluorescence microscopy as compared to conventional microscopy (Figure S3c-e) was ascribed to the increased sensitivity of fluorescence microscopy.

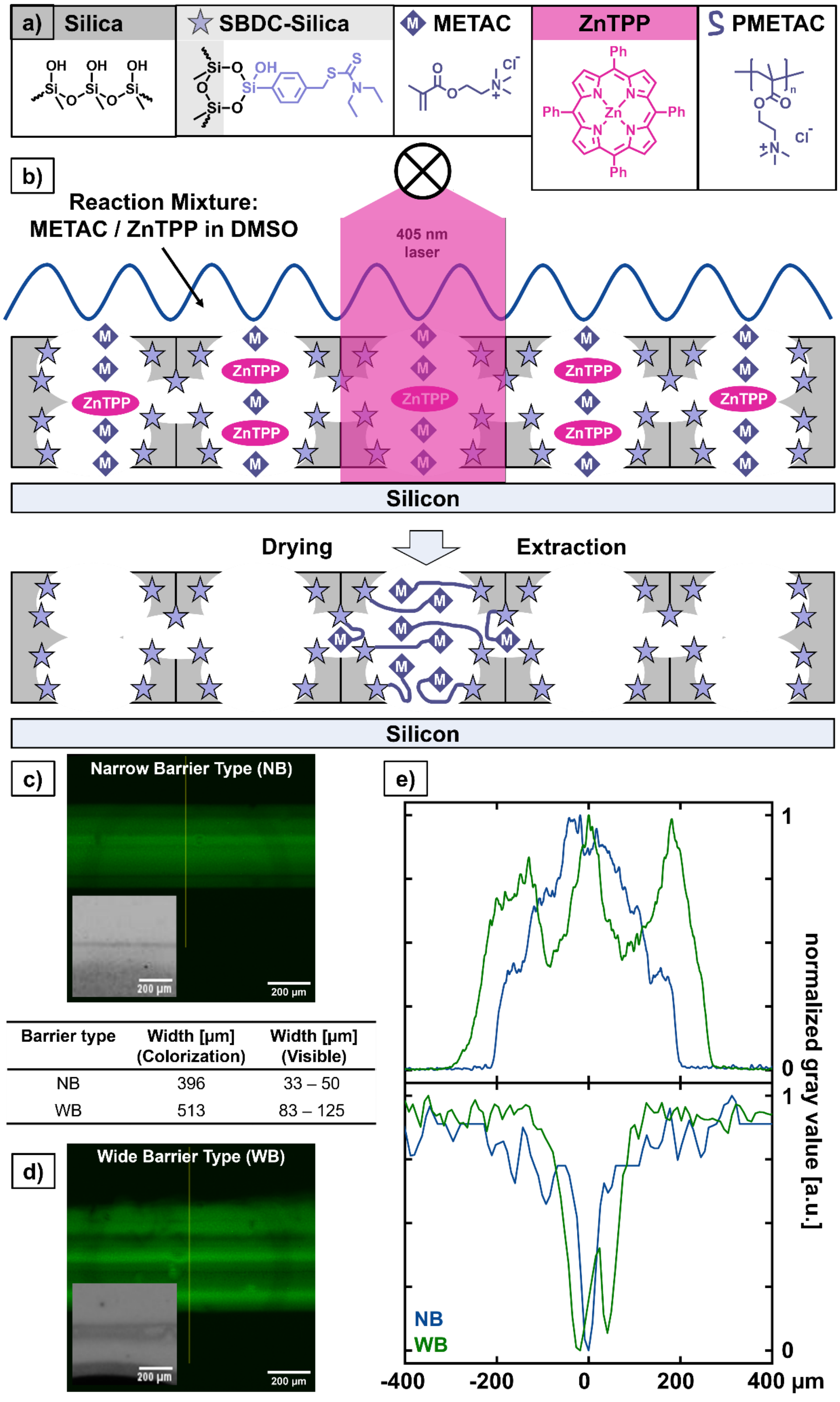

Barrier type	Width [µm] (Colorization)	Width [µm] (Visible)
NB	396	33 – 50
WB	513	83 – 125

Figure S3. a) Chemical structure of reactants involved in surface-initiated PET-RAFT polymerization of META-Cl in MPS thin-films, leading to PMETA-Cl; b) schematic illustration of DLW mediated PMETA-Cl formation in the beam path of a 405 nm laser, showing the resulting MPS-polymer hybrid material; imaging of the studied c) narrow barrier (NB) and d) wide barrier (WB) types (Figure S4) using fluorescence microscopy upon fluorophore adsorption (big picture) as well as a conventional camera (small picture inset), respectively; e) gray value (GV) profile along the barrier widths of NB and WB types, determined from the fluorescence micrographs (upper chart) and optical imaging (lower chart), respectively.

4. Determination of Visible Barrier Widths

To characterize PMETA-Cl barriers, the visible barrier widths were determined through Gauss modeling of the respective barrier GV profiles (experimental section), describing local changes in refractive index between the laser-written polyelectrolyte line and the unfunctionalized MPS thin film. The Gauss modeling was deemed appropriate as the laser intensity profile shows a Gaussian shape. To evaluate potential deviations in barrier width, the barrier widths were calculated along the entire barrier length for all barrier sections subjected to liquid imbibition. Figure S4a shows the so-obtained barrier widths assigned to the individual experiments, showing barrier widths of approximately 33 – 50 µm for the NB type (15 mW initial laser power during DLW) and 83 – 125 µm for the WB type (25 – 33 mW initial laser power during DLW). The correlation between laser power and barrier width seems to be limited to the lower irradiation powers 15 and 25 mW whereas a further increase of laser power to 33 mW no longer results in a substantial expansion of barrier widths, pointing at a limited extent of active species diffusion during the polymerization under these conditions. Consequently, similar barrier widths are obtained for both laser powers. Due to a lower contrast of the thinner barriers to the background, which becomes particularly evident for NB2, the Gauss fitting was less precise for the NB type, as partially leading to elevated standard deviations in the determination of barrier widths. Deviations in width along the barrier are considered to be manufacturing-related, most probably resulting from optical imperfections of the glass Petri dish used as reaction vessel. In this context, an inhomogeneous glass surface may induce defocusing of the laser beam, hence explaining the barrier broadening especially observed for WB2. Slightly tilted substrate placement may induce additional beam expansion or contraction, potentially increasing the inhomogeneities in barrier widths. Nevertheless, the measured barrier widths remain comparable for the NB and WB types, respectively, with the observed differences mostly lower or comparable to the standard deviation. According to method 1 (experimental section), deviant barrier widths of 83.1 µm (Figure 3a) and 112.7 µm (Figure 3b) were calculated for WB2 which exhibits a more distinct appearance at the head of the PMETA-Cl line (Figure S4b) in contrast to a more diffuse appearance at the tail of the PMETA-Cl line (Figure S4c). The differing manifestation of the observed diffuse fringes, being more distinct in Figure S4c at a GV of 54% of the minimum value, leads to significant differences in Gauss modelling, generating a narrower profile for the more distinct barrier and a broadened profile for the more diffuse barrier section to which the higher width is assigned. Hence, the less significant diffuse fringes of are improperly overweighted against the barrier core at minimum GV values, where according to the progression of the refractive index the highest local polymer densities are expected. To describe the core section of the barrier more accurately, the Gauss modeling process is adjusted, performing method 2 at a fixed amplitude.

a) Figure	Salt	Barrier	Width [µm] (method 1)		Width [µm] (method 2)	
2a	$BaCl_2$	NB1	39.4	± 7.7	-	-
2a	$BaCl_2$	WB1	105.9	± 3.7	-	-
2b	$MgSO_4$	NB1	49.1*	± 8.3*	-	-
2b	$MgSO_4$	WB1	125.0	± 8.0	-	-
2c	NaCl	NB1	45.9*	± 8.3*	-	-
2c	NaCl	WB1	118.0	± 14.1	-	-
2d	$MgCl_2$	NB1	33.5	± 9.4	-	-
2d	$MgCl_2$	WB1	107.3	± 7.6	-	-
3a	$MgSO_4$ (1st)	WB2	83.1*	± 7.6*	69.8	± 6.2
3b	$BaCl_2$ (1st)	WB2	112.7	± 12.6	67.9	± 7.0
SI 5-1	$BaCl_2$	NB2	46.4*	± 15.7*	-	-
SI 5-2	$MgSO_4$	NB2	66.1**	± 18.1**	-	-
SI 5-3	NaCl	NB2	49.2*	± 13.8*	-	-
SI 5-4	$MgCl_2$	NB2	46.8	± 16.4	-	-

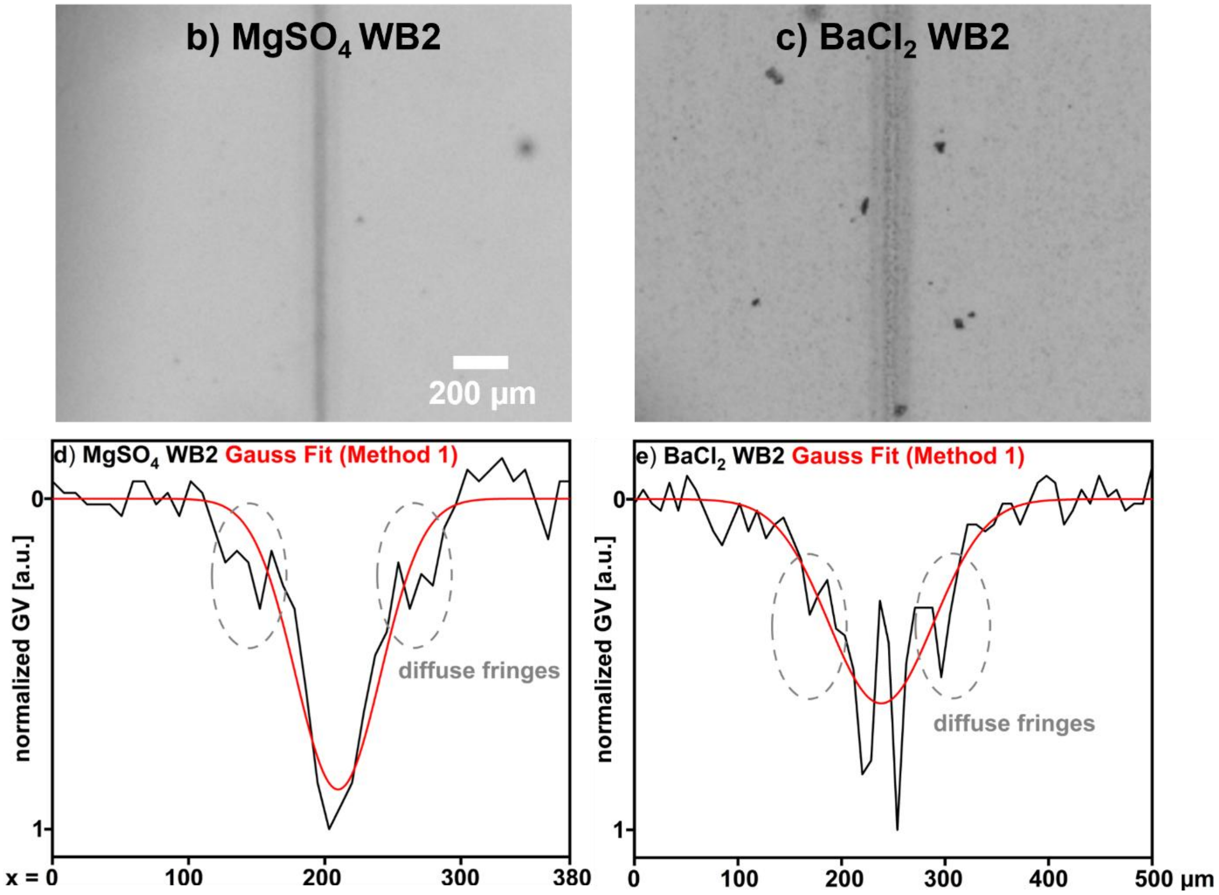

Figure S4. a) Width of all barrier section calculated through Gauss modeling (experimental section); images of WB2 in the section of b) $MgSO_4$ and c) $BaCl_2$ solution imbibition (Figure 3a-b); WB2 GV profile (black) and the corresponding Gaussian (red) for the section of d) $MgSO_4$ and e) $BaCl_2$ solution imbibition (Figure 3); *) to increase the quality of the Gaussian fits, the underlying GV profiles were smoothed in sections; **) due to the low image contrast, the Gaussian fit quality was significantly reduced.

Thereby, comparable averaged barrier widths of 69.8 µm (Figure 3a) and 67.9 µm (Figure 3b) are determined, confirming the similar barrier core structure of both barrier sections for which due to the elevated polymer densities the strongest polymer-ion interactions are expected. In general, consistent laser powers during DLW are expected to afford similar polymer amounts for a single barrier regardless of deviations in barrier width which inherently supports the comparability of barriers prepared in a single run.

5. Barrier-Regulated Imbibition of Aqueous Salt Solutions through the NB Type

a)

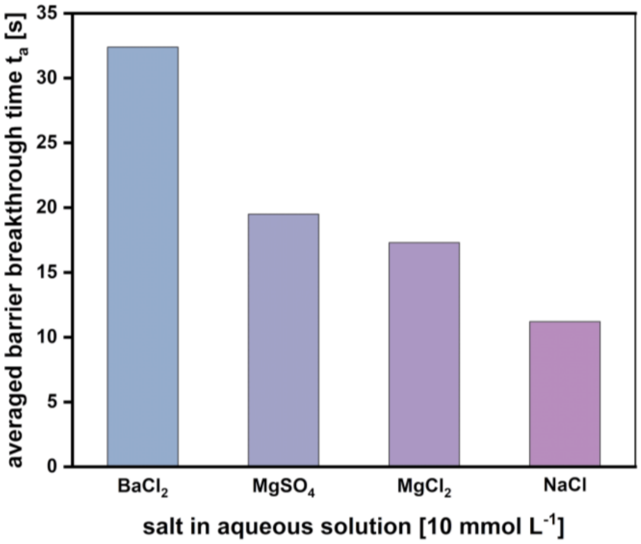

b) Exp.	Salt	DBD [µm]	Barrier Breakthrough Time t_a [s]			Free Imbibition Width at 189 s [µm]
		NB2	NB1	NB2	Avg.	
1	$BaCl_2$	237.6	39.3	23.5	32.4	534.9
2	$MgSO_4$	306.9	25.5	13.5	19.5	435.0
3	NaCl	220.5	18.2	4.2	11.2	567.0
4	$MgCl_2$	260.5	18.5	16.1	17.3	527.6

Figure S5. a) Averaged salt-specific barrier breakthrough times ta in interaction with NB1 and NB2, prepared under identical conditions; b) comparison of t_a values in interaction with NB1 and NB2 in relation to droplet-NB2 distances and salt-specific imbibition widths at 198 s after droplet deposition.

To confirm the previously observed trend of salt-specific barrier permeation, NB-regulated imbibition experiments of aqueous $BaCl_2$, $MgSO_4$, NaCl, and $MgCl_2$ solutions were repeated with the focus on salt-specific barrier breakthrough times t_a which were compared to the results in Figure 2. Given the similar properties of NB1 (Figure 2) and NB2 (Figure S5) and the comparable experimental parameters (salt concentration, climate conditions, DBD set according to Figure 2), the obtained t_a values were averaged to describe the salt-specific permeation through the PMETA-Cl barrier more comprehensively. Figure S5a depicts the averaged t_a of all salt solutions studied while Figure S5b shows the detailed comparison of t_a values in interaction with NB1 and NB2. The variation of t_a indicates experiment-specific droplet-NB2 distances and salt-specific imbibition widths influences at a given time.

Nevertheless, the trend seems to be reproducible. Consistent with Figure 2 and corresponding to the differing cation properties of Na^+ and Ba^{2+}, NaCl solution exhibits the lowest barrier breakthrough time of 4.2 s while $BaCl_2$ solution exhibits the highest barrier breakthrough time of 23.5 s. Despite identical process parameters, a slightly more diffuse laser beam or a marginally tilted substrate placement during the barrier preparation may explain the slightly elevated barrier widths (Figure S4a) for NB2 which are probably accompanied by lower PMETA-Cl peak densities inside the mesopores, as potentially facilitating permeation through NB2 at a lower t_a. Again, $MgSO_4$ and $MgCl_2$ solutions yield intermediate t_a values, emphasizing the facilitated barrier permeation of Mg^{2+} in contrast to Ba^{2+} ions. However, contrarily to Figure 2, a faster barrier permeation of $MgSO_4$ than of $MgCl_2$ solution is observed in Figure S5. In conclusion, Figure S5 confirms the salt-specific trend of barrier breakthrough times, indicating that cation properties are the most significant limiting factor affecting barrier permeation.

6. Permeation of Aqueous $MgSO_4$ Solution through the PMETA-Cl Barrier

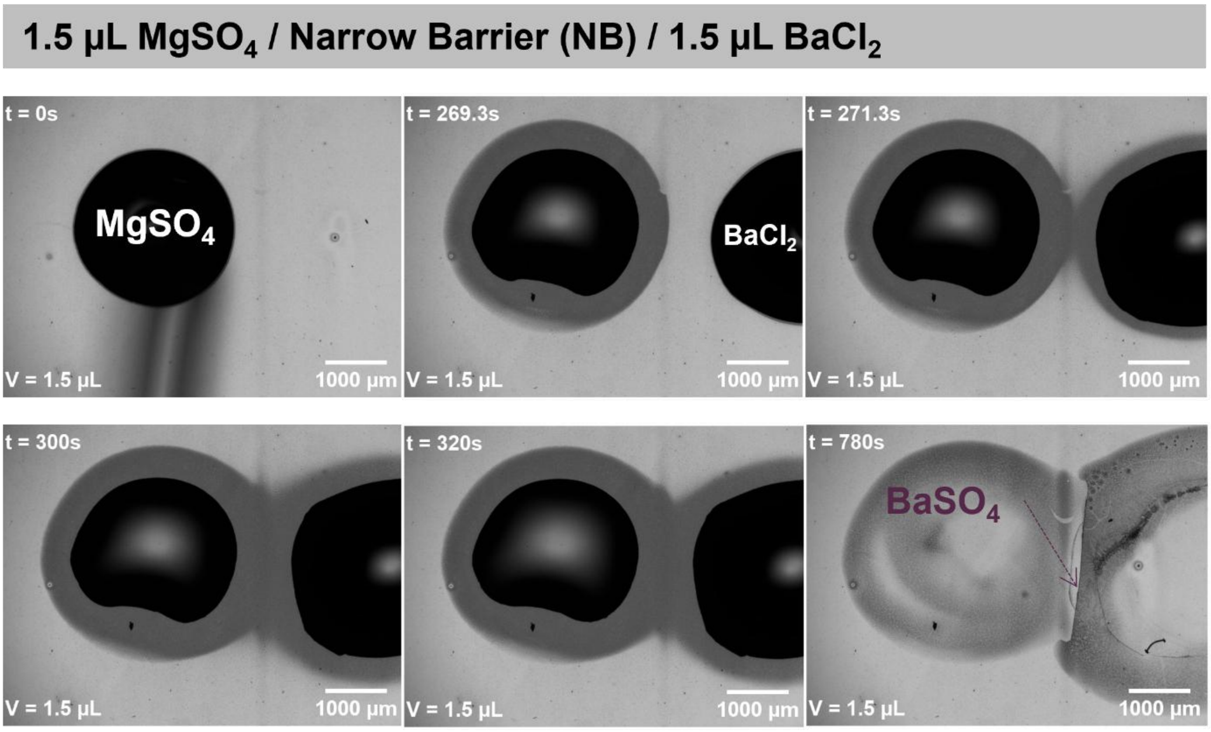

Figure S6. NB-regulated imbibition of aqueous $MgSO_4$ solution and communication between droplets of 1) aqueous $MgSO_4$ solution and 2) aqueous $BaCl_2$ solution which are separated by the barrier: imaging of the PMETA-Cl barrier-regulated imbibition and droplet communication process.

To verify the transport of SO_4^{2-} ions through the NB type, a droplet of aqueous $BaCl_2$ solution was deposited opposite of the NB upon barrier breakthrough of the imbibition front corresponding to the initially deposited $MgSO_4$ solution droplet. Figure S6 shows the barrier crossing of the imbibition front of a 1.5 µL droplet of $MgSO_4$ solution. The low area of the imbibition ring extension across the barrier

to the elevated DBD of approximately 417 µm, precluding a more substantial extension of the imbibition ring due to the decelerated imbibition advancement characteristic for $MgSO_4$ solution (Figure 2). Nevertheless, substantial formation of $BaSO_4$ precipitate is observed upon rapid merging of both annuli which becomes particularly distinct after full solvent evaporation. The formation of $BaSO_4$ precipitate at the contact line of both annuli demonstrates the transport of SO_4^{2} ions through the PMETA-Cl barrier which are essential for precipitate formation. Concomitant permeation of Mg^{2+} counterions, necessary to maintain the charge balance in the imbibition zone, is presumed. The formation of precipitates shows the permeability of the NB type to SO_4^{2-} ions and contradicts substantial ion retention/filtering effects through the barrier.

7. Permeation of Aqueous BaCl2 Solution through the PMETA-Cl Barrier

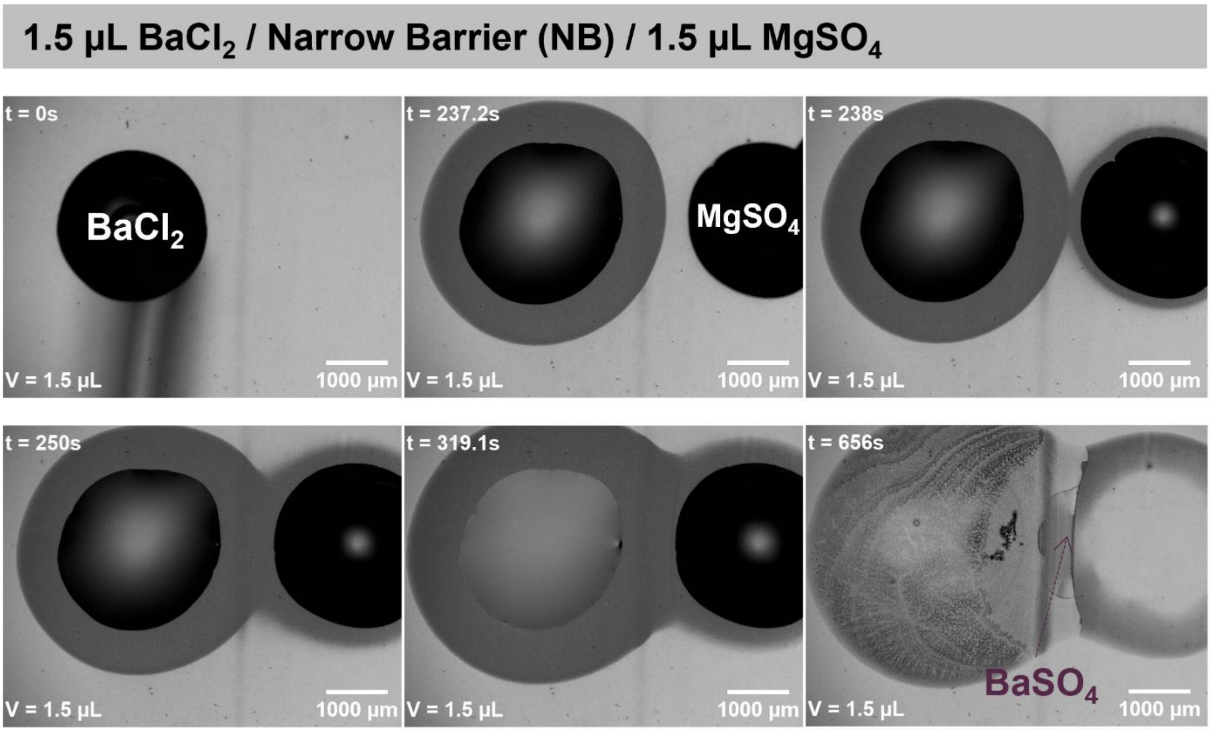

Figure S7. NB-regulated imbibition of aqueous $BaCl_2$ solution and communication between droplets of 1) aqueous $BaCl_2$ solution and 2) aqueous $MgSO_4$ solution which are separated by the barrier: a) Imaging of the PMETA-Cl barrier-regulated imbibition and droplet communication process.

To show the transport of Ba^{2+} ions through the NB type, a droplet of aqueous $MgSO_4$ solution was deposited opposite of the NB upon barrier breakthrough of the imbibition front corresponding to the initially deposited $BaCl_2$ solution droplet. Figure S7 depicts the significant barrier crossing of the imbibition front related to $BaCl_2$ solution. Upon placement of the $MgSO_4$ solution droplet beyond the PMETA-Cl barrier, rapid merging of the imbibition annuli occurs within 0.8 s. The subsequently emerging line of $BaSO_4$ precipitate at the contact line of both imbibition rings, which becomes particularly visible after full solvent evaporation, clearly indicates the transport of Ba^{2+} of through the

PMETA-Cl barrier which are essential for precipitate formation. It demonstrates the permeability of the NB type to Ba^{2+} ions while contradicting substantial ion retention effects through the barrier observed in Figure 3b.

8. Relationship between Droplet Volumes and Imbibition

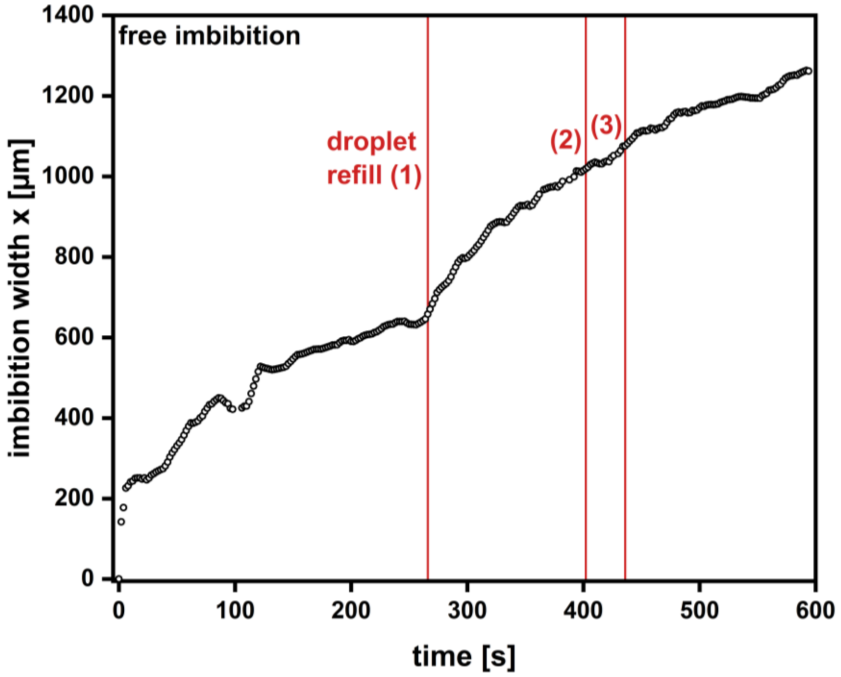

Figure S8. Free imbibition advancement of $BaCl_2$ upon droplet replenishment in the studied MPS thin films; the red lines indicate the time of droplet replenishment in successive steps of 1.5 µL (the imbibition process was initiated by initial deposition of a 1.5 µL $BaCl_2$ solution droplet at t = 0s.

To assess the influence of droplet replenishment and volume increase, used to increase the droplet lifetime and thus the imbibition times in Figure 3, on imbibition advancement, free imbibition of $BaCl_2$ solution was tracked during successive droplet replenishment (Figure S8), as leading to volume increase. As already observed for WB-regulated imbibition (Figure 3), the free imbibition rates apparently moderate toward zero before the first droplet replenishment (Figure S8), indicating the transition into steady state imbibition dynamics at prolonged times also observed for WB-regulated imbibition (Figure 3a, step 2-3). The first droplet replenishment immediately results in increasing imbibition rates accompanied by an expansion of the droplet perimeter by 162.7 µm toward the imbibition front (Figure S8). According to Darcy's law,[7] the increased imbibition rates presumably correspond to an increased solution volume flow which is inversely proportional to the distance between the droplet perimeter and the imbibition front, reduced by 162.7 µm as consequence of droplet replenishment. The increased imbibition rates at $t > 266$ s may be further enhanced by pore condensation promoted in closer proximity to the droplet perimeter.[8] In contrast, the second and third droplet replenishments, which are

accompanied by droplet expansion in directions away from the imbibition front and thus constant distances between the droplet perimeter and the imbibition front, do not induce a measurable increase in imbibition rates, with the rates instead further moderating toward the expected steady state. Figure S8 clearly shows that the volume increase of the droplet only influences imbibition rates when the droplet expands toward the imbibition front, thereby reducing the distance between the droplet perimeter and the imbibition front.

9. Laser Power Measurements

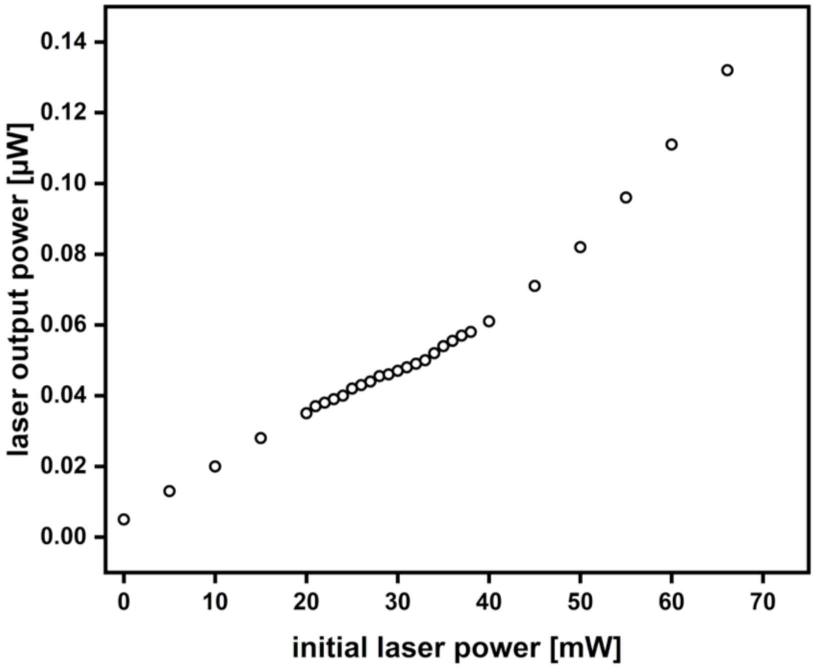

Figure S9. Measurement of the laser output power obtained from the 405 nm laser.

To quantify the light power to which the mesoporous matrix is exposed during DLW-mediated polymerization, the laser output power exiting the device was determined. Figure S9 relates the initial laser power to the emitted laser output power, showing a significant decay in power occurring along the light path of the laser beam. The power loss is ascribed to reflection, beam deflection and partial absorption processes within the optical components of the device. Despite the reduced laser output power, sufficient polymerization is still achieved at low irradiations energies of 30 nJ (NB) and 40 nJ (WB) per spot, emphasizing the low energy demand of the PET-RAFT polymerization employed for DLW.

References

[1] D. Richter, A. Andrieu-Brunsen, “Laser-Writing of Fluorescent Copolymers in Mesoporous Silica Thin Films,” *Advanced Materials Interfaces* 11, no. 36 (2024): 2400538. https://doi.org/10.1002/admi.202400538.

[2] M. Stanzel, U. Kunz, A. Andrieu-Brunsen, “Layer-selective functionalisation in mesoporous double layer via iniferter initiated polymerisation for nanoscale step gradient formation,” *European Polymer Journal* 156 (2021): 110604. https://doi.org/10.1016/j.eurpolymj.2021.110604.

[3] A. D. Pizarro, C. L. A. Berli, Soler-Illia, Galo J. A. A., M. G. Bellino, “Ion–Fluid Transport-Control Feedback along Nanopore Networks,” *ACS Nano* 18, no. 25 (2024): 16199. https://doi.org/10.1021/acsnano.4c01898.

[4] M. Mercuri, K. Pierpauli, M. G. Bellino, C. L. A. Berli, “Complex Filling Dynamics in Mesoporous Thin Films,” *Langmuir* 33, no. 1 (2017): 152. https://doi.org/10.1021/acs.langmuir.6b03987.

[5] C. Förster, R. Lehn, A. Andrieu-Brunsen, “Automated Multi- and Block-Copolymer Writing in Mesoporous Films Using Visible-Light PET-RAFT and a Microscope,” *Small* n/a, no. n/a (2023): 2207762. https://doi.org/10.1002/smll.202207762.

[6] C. Förster, R. Lehn, E. M. Saritas, A. Andrieu-Brunsen, “Laser Writing of Block-Copolymer Images into Mesopores Using SBDC-Initiated Visible-Light-Induced Polymerization,” *Angewandte Chemie International Edition* 62, no. 19 (2023): e202217806. https://doi.org/10.1002/anie.202217806.

[7] S. Gruener, P. Huber, “Imbibition in mesoporous silica: rheological concepts and experiments on water and a liquid crystal,” *Journal of Physics: Condensed Matter* 23, no. 18 (2011): 184109. https://doi.org/10.1088/0953-8984/23/18/184109.

[8] J. Trosseille, H. Bellezza, O. Vincent, “Coupled imbibition and evaporation of droplets deposited on a nanoporous layer,” *arXiv preprint arXiv:2510.19932v1, 2025*.
